# IFNγ and TNFα optimize salivary gland mesenchymal stromal cells: an alternative to marrow- and adipose-MSCs for radiation xerostomia

**DOI:** 10.1016/j.reth.2025.11.004

**Published:** 2025-11-14

**Authors:** Michele C. Larsen, Ilya Gurevic, Liliana Berube, Addie Vande Loo, Elizabeth Hanson, Ryan Adam, Valeria Manfrè, Maxwell Parker, Cristina Paz, Jacques Galipeau, Randall J. Kimple, Grace Blitzer, Sara S. McCoy

**Affiliations:** a Department of Medicine, School of Medicine and Public Health, Division of Rheumatology University of Wisconsin, Madison, WI, USA; b Department of Human Oncology, School of Medicine and Public Health, Madison, WI, USA; c Department of Medicine, University of Udine, Academic Hospital “Santa Maria Della Misericordia,”, Udine, Italy; d Department of Medicine, University of Wisconsin Carbone Comprehensive Cancer Center, University of Wisconsin, Madison, WI, USA; e Department of Medical Physics, School of Medicine and Public Health, University of Wisconsin, Madison, WI, USA

**Keywords:** Mesenchymal stromal cell, Salivary gland, Bone marrow, Adipose, Radiation, Xerostomia, Secretome, IFNγ, TNFα, Cytokine

## Abstract

**Objectives:**

Local mesenchymal stromal cell (MSC) administration is a promising therapy for xerostomia. MSCs deploy their advantageous effects through their trophic secretome and immunomodulatory capabilities. These functions are enhanced with IFNγ pre-licensing, but the effects of TNFα pre-licensing are unknown. Our objective was to compare MSCs by tissue source (MSC(BM), MSC(AD), and salivary gland-derived [MSC(SG)]) and by cytokine pre-licensing conditions.

**Methods:**

We used single cell and bulk RNA sequencing and ELISA to determine key trophic and immunomodulatory features differing between human MSC(BM), MSC(AD), and MSC(SG). We used ELISA and flow cytometry of T-cell co-culture to define the effect of IFNγ and/or TNFα on MSC trophic secretome and immunomodulatory capacity. Finally, we studied salivary flow and glandular recovery with MSC injection in radiation-induced xerostomia mice.

**Results:**

Bulk RNA sequencing (RNAseq) of MSC(BM), MSC(AD), and MSC(SG) revealed that they shared 85 % of transcripts. Key differences included extracellular matrix production and response to cytokines in MSC(SG). Single cell RNA sequencing showed MSC(SG) treated with IFNγ and TNFα transcriptionally diverged from other treatment conditions. Regardless of MSC source, dual stimulation of MSCs with IFNγ and TNFα produced an average of more than a 20-fold increase in R-Spondin 3 compared to vehicle conditions. Additionally, IFNγ and TNFα pre-licensing optimized immunomodulatory marker expression more than IFNγ alone. Intercellular adhesion molecule 1 increased 12-fold more, programmed death ligand 1 increased 1.4-fold more, and indoleamine 2,3 dioxygenase increased 2-fold more with IFNγ/TNFα pre-licensing than IFNγ alone. Both cytokine stimulation conditions resulted in a 1.2-fold decrease in T-cell proliferation. Gland structure, aquaporin 5, and salivary flow are preserved in irradiated mice treated with MSC(SG) pre-licensed with IFNγ/TNFα.

**Conclusion:**

MSC(SG) pre-licensed with both IFNγ and TNFα deploy advantageous functional cell attributes for salivary gland regenerative medicine.

## 1 Introduction

Xerostomia is the subjective sensation of dry mouth and occurs in 30–40 % of patients who receive radiation for head and neck cancer [1]. Patients with xerostomia are at risk for developing dental caries, impaired swallowing ability, difficulty speaking, diminished taste, and poor nutritional status. Ultimately, these symptoms result in a three-fold reduction of oral health-related quality of life [2]. Therapy for patients with radiation-mediated xerostomia from head and neck cancer is symptomatic and includes approaches such as topical wetting, saliva stimulation with chewing gum and candies, or use of cholinergic agents. These symptom-targeted therapies have limited efficacy and high rates of side effects. There is a major need for novel therapies that address the underlying pathogenesis to alleviate xerostomia in these patients.

Mesenchymal stromal cells (MSCs) reside in tissue stroma and impart potent immunomodulatory properties. MSCs expand regulatory T cells and suppress cytotoxic T cells, processes mediated through key proteins including indoleamine 2,3-dioxygenase (IDO), Programmed Death-Ligand 1 (PD-L1), and Intercellular Adhesion Molecule 1 (ICAM-1) [[3], [4], [5]], among others. In addition to their immunomodulatory properties, MSCs also have trophic effects and support local progenitor niche cells. Marrow-derived MSCs (MSC(BM)) sustain LGR5+ epithelial stem cells in the gut via expression of Wnts, fibroblast growth factors (FGFs) and R-Spondins [6]. MSC(BM) accelerate intestinal epithelial recovery following sub-lethal total-body radiation, likely through their secretome including Wnt2b and R-Spondin [7].

The immunomodulatory effects of MSCs are amplified with in vitro IFNγ pre-stimulation (or pre-licensing) [[8], [9], [10], [11]]. Additionally, IFNγ treatment prior to cryopreservation improves post-thaw recovery and functional characteristics of MSCs, a critical advantage for therapeutic MSCs utilized more broadly [[8], [9], [10]]. Recent studies established that dual stimulation with both IFNγ and TNFα synergistically enhances the immunomodulatory capacity of MSCs [[12], [13], [14], [15]]. Intact immune response is pivotal for epithelial wound healing post irradiation. Radiation causes local immune injury, including depletion of epithelial-associated macrophages, which are required for post-radiation tissue repair and gland function [[16], [17], [18]]. Additionally, though total CD3^+^ T cells are depleted, the CD3^+^CD4^+^CD8^+^ subpopulation of T-cells is increased [18,19]. One existing hypothesis is that chronic T-cell infiltrate and the resultant inflammatory cytokines promote loss of salivary gland function; however, little is known about the potential mechanisms behind this phenomenon [19]. Other mechanisms implicated in radiation induced salivary gland damage include apoptosis and senescence or dysfunction of progenitor cells, indicating trophic factors might also mitigate radiation-induced damage [20]. The effects of dual cytokine stimulation on the trophic secretome of MSCs remain unknown.

The unique and potent properties of MSCs make them a promising low risk therapy to alleviate fibrotic and inflammatory diseases. Accordingly, MSCs have recently been deployed in studies as a treatment for radiation-induced xerostomia. These studies use adipose-derived MSCs (MSC(AD)) or IFNγ pre-licensed MSC(BM) [21,22] with encouraging initial results. Despite promising therapeutic results, the process of obtaining MSC(BM) and MSC(AD) can be associated with pain or other adverse effects. Bone marrow aspirates result in 4 % of patients experiencing unbearable pain and 32 % of patients experiencing severe pain [23,24]. Liposuction requires general anesthesia and is associated with major complications such as aspiration pneumonia, anesthesia reaction and cases of pneumothorax or death [25,26]. In contrast, the labial salivary gland (SG) biopsy, from which MSC(SG)s are collected, is well tolerated, requiring only local lidocaine prior to a <1 cm incision in the inner lower lip [27]. Post-procedure care consists of the low-risk regimen of ice and NSAIDs. No major organ or life-threatening risks are associated with this minor and relatively low-cost procedure. Finally, SGs might represent a better source of MSCs for xerostomia because its source tissue aligns with the therapeutic target tissue.

Our objective is to compare MSC(SG) to more traditional MSC sources including MSC(AD) and MSC(BM), evaluating the effects of cytokine stimulation (IFNγ and TNFα) on the trophic secretome and function of MSC(SG) in the treatment of radiation-induced xerostomia.

## 2 Methods

### 2.1 Human samples

All the work described was performed under the University of Wisconsin Health Sciences IRB 2016–1545, 2018–0815 and 2022–1491 and are completed in accordance with the Declaration of Helsinki. Informed consent was obtained for all human subjects. MSC(AD) were obtained from intra-abdominal adipose tissue and MSC(BM) were sourced from bone marrow as previously described [28] and were derived from deidentified healthy donors. MSC(SG) were obtained from labial SG biopsies as previously described [27,29].

SG epithelial cells and were cultured via explant outgrowth methodology as described in the Supplemental Methods.

To ensure our isolated cells were MSCs, our cells met criteria as per the International Society of Cell Therapy including adherence to plastic, differentiation capability (Supplemental Fig. 1A), and surface markers [[29], [30], [31], [32]]. Furthermore, all MSCs were evaluated to ensure longer cryopreservation did not negatively impact doubling time of MSC(AD) and MSC(BM), and they were viable (Supplemental Fig. 1B–D). Differentiation assays were performed using R&D Stem X Vivo adipogenic and osteogenic differentiation supplements, in accordance with manufacturer's protocols.

Demographics were not available for the deidentified MSC(BM) and MSC(AD) healthy donors. Demographics for MSC(SG) are shown in Table 1. Labial SG donors classified as healthy controls volunteered to donate labial SGs prior to radiation for head and neck cancer. Labial SGs were not involved in any active disease process. Controls had dry eye and/or mouth symptoms so proceeded with labial SG biopsy, but they did not have any evidence of an autoimmune process driving their symptoms (based on labs and pathology). We termed these patients sicca (meaning symptoms of dryness) controls. Because we were interested in using MSCs to treat other causes of xerostomia in the future, we also collected MSCs from Sjögren's disease (SjD) subjects met 2016 ACR/EULAR criteria for disease [33].

**Table 1:** Demographics.

	HC	Sicca	SjD
	n = 4	n = 3	n = 3
Age mean (SD)	51 (4)	45 (13)	52 (5)
Female n (%)	1 (33)	3 (100)	3 (100)
Race (european) n (%)	3 (100)	3 (100)	2 (66)
SjD feature n (%)			
Positive SSA antibody			3 (100)
Low complement			1 (33)
High RF			2 (66)
Labial SG focus score			1.6 (1.4)

HC = healthy control; Sicca = dryness symptoms but no evidence of immune activity; SjD = Sjögren's disease.

SD = standard deviation.

### 2.2 Doubling time and viability assays

Doubling time (Td) was calculated: Td = T (h) x ln2/ln (final cell count/initial cell count). Metabolic ATP production was measured using the CellTiter Glo 2.0 viability assay kit (Promega, Madison, WI). Luminescence was recorded using a Biotek Synergy 2 plate reader. Viability was quantified relative to an ATP standard curve. Additional details are provided in the Supplemental Methods.

### 2.3 RNA-seq

Sixteen populations of cells (n = 3 MSC [BM]; n = 3 omental MSC [AD]; n = 3 sicca MSC [SG]; n = 3 SjD MSC [SG]; and n = 4 non-sicca MSC [SG]) were seeded at ∼3–5K/cm^2^ for BM, ∼7–10 K/cm^2^ for adipose and ∼1–4K/cm^2^ for SG-derived MSCs. After reaching 80 % confluence, MSCs were serum starved for 24 h. No cytokine treatment was performed. RNA isolation and purification was performed using the DirectZol RNA Miniprep Plus Kit (ZymoResearch, Irvine, CA) according to the manufacturer's instructions. The isolated RNA was provided to Novogene, Inc (Sacramento, CA) for sequencing using the Illumina platform. Briefly, Novogene isolated messenger RNA from total RNA with poly-T-oligo-attached magnetic beads. The library was constructed after fragmentation and cDNA synthesis and after end repair, A-tailing, size selection, adaptor ligation, amplification, and purification. A standard bioinformatic pipeline was deployed by data QC (error rate distribution, GC-content distribution, and data filtering), mapping to reference genome, gene expression quantification, and differential expression analyses (https://doi.org/10.5061/dryad.9ghx3ffv6). Novogene performed functional analysis including enrichment analysis.

### 2.4 Single cell RNA sequencing

Single cell RNA (scRNA) sequencing was performed on three untreated clinically healthy control MSC(SG) biological replicates. Each replicate was treated with vehicle, IFNγ (60 ng/mL), TFNα (10 ng/mL), or dual cytokine (IFNγ 60 ng/mL and TNFα 10 ng/mL) for 24 h before cryopreservation as above (https://doi.org/10.5061/dryad.9ghx3ffv4). High quality cell suspensions were prepared with an average viability of 91 % and loading concentration of 1181 cells/μL. scRNA-seq libraries were constructed using PIPseq T2 3′ Single Cell RNA Kit v4.0PLUS, following the manufacturer's protocol. Libraries were sequenced on an Illumina NovaSeq X sequencer with a sequencing depth of at least 20,000 reads per cell. Gene-barcode matrices were created using the PIPseeker program, which utilized STAR to map reads to the human genome (GRCh38. p13). Cells with less than 1000 and more than 3000 genes detected were excluded, as well as cells with less than 500 and more than 5000 UMI counts, and more than 10 % of the transcripts from mitochondrial genes [34]. Genes detected in less than five cells were also filtered out. Downstream analysis was performed using R (version 4.1.2) with the Seurat package (version 4.40), including merging datasets, normalizing, scaling, and running PCA. The Harmony package (version 1.2.3) was used to correct for batch effects before performing clustering and UMAP visualization. Differential gene expression analysis was performed between treatments and vehicle control to identify markers for each condition, filtering to include only upregulated genes expressed in at least 10 % of the cells within a treatment with at least a 1.23-fold change in expression. Pathway analyses were performed with 2024 Reactome through Enrichr using only transcripts with a q-value (aka adjusted p-value) of ≤0.05 [[35], [36], [37]].

### 2.5 ELISA

To establish the trophic secretome, MSC(BM) (n = 3), MSC(AD) (n = 3), and MSC(SG) (n = 9) were treated with IFNγ (10 ng/mL)); PeproTech, Cranbury, NJ), TNFα (10 ng/mL; BioLegend, San Diego, CA), or TGFβ (2.5 ng/mL; PeproTech, Cranbury, NJ). After 48 h of culture, the conditioned media was used for ELISA per the manufacturer's recommendations (GDNF [Innovative Research Novi MI], RSPO1 [CUSABIO, Houston, TX], RSPO3 [Innovative Research], Wnt1 [CUSABIO], Wnt2b [CUSABIO], Wnt3a [CUSABIO], Wnt4 [Raybiotech, Norcross, GA], Wnt5a [CUSABIO]) with dilutions described in the Supplemental Methods.

### 2.6 Organoid generation and quantification

Organoid culture media was prepared by supplementing DMEM (40 %)/F12 (60 %) with 1 % Glutamax, 1 % N2, 20 ng/mL EGF, 20 ng/mL FGF-2, 10 μg/mL human insulin, 1 μM dexamethasone, 10 μM Y-27632, 100 ng/mL Wnt3a, 12.5 ng/mL noggin, 1 % penicillin/streptomycin [38,39]. The epithelial cells were resuspended in diluted Matrigel (Corning, 354,248). Organoids were cultured for five days either without supplementation or supplemented with 500 ng/ml R-Spondin 1 or 500 ng/ml R-Spondin 3. Organoid cultures were imaged daily using an Olympus CK40 light microscope, selecting an optical field in each Matrigel dome that contained at least 10 organoids. Q-path analysis of individual organoid area was calculated for 10 organoids per image. The data represents the average area (p^2) (±SEM) for the 20 organoids per treatment resultant from two individual wells.

Immunohistochemistry (IHC) and immunofluorescence (IFC) analysis was completed on organoids cultured in the organoid culture media described above that was supplemented with R-Spondin 1. Standard H&E methods were used as described in the Supplemental Methods. IFC was performed using fluorochrome-conjugated antibodies for Keratin 5 (Krt5-AF647) (1:100, Abam), Keratin 7 (Krt7-AF594) (1:250, Biolegend), and Keratin 14 (Krt14-FITC) (1:100, EMD Millipore) and Aquaporin 5 (Aqp5-AF647) (1:500, Santa Cruz Biotechnology) (Supplemental Table 1).

### 2.7 Flow cytometry

After culture, treatment with vehicle, 60 ng/mL IFNγ and/or 10 ng/mL TNFα, cryopreservation, and cryorecovery as previously described, MSCs (200 K/per sample) were stained with the following fluorochrome-conjugated antibodies: Indoleamine 2,3-dioxygenase (IDO)-FITC (1:100, Invitrogen), CD54 (ICAM-1)-APC (1:100, Miltenyi Biotec) and CD274 (PD-L1)-BV421 (1:100, BD Biosciences). Intracellular staining of IDO was completed using the eBiosciences intracellular fixation and permeabilization buffer set, in accordance with manufacturer's instructions. The PBMCs were stained with Ki67-PE (1:100, BD Biosciences) and CD3-FITC (1:100, BD Biosciences). Intracellular Ki67 staining was completed using the eBiosciences FoxP3 fixation/permeabilization kit, in accordance with the manufacturer's protocol. Live/Dead cell populations were assessed using Ghost Red 780 viability dye (Tonbo Biosciences). Additional details are provided in the Supplemental Methods.

### 2.8 MSC-PBMC co-culture

MSCs were treated with vehicle, 60 ng/mL IFNγ and/or 10 ng/mL TNFα prior to cryopreservation as described above. The cells were plated to a 96-well plate (6 K cells/well) and cultured for 24 h prior to the addition of healthy allogeneic PBMCs. Cryopreserved PBMCs were slowly thawed by the addition of media to the cells. The cells were washed and directly added to the MSC cultures in a 10:1 ratio of PBMCs to MSCs. The cells were stimulated with 2 μL/ml PHA-L (eBioscience). The co-culture remained undisturbed for a further 4 days. The non-adherent PBMCs were collected, and T-cell proliferation was analyzed by flow cytometry. MSCs were quantified using CellTiter Glo 2.0 assay per the manufacturer's recommendations (Promega) and T-cell proliferation was normalized to total PBMCs in the co-culture to account for variation in MSC density affecting immunosuppressive properties on MSCs.

### 2.9 Mouse MSC isolation and culture

All animal experiments were approved by the University of Wisconsin-Madison Institutional Animal Care and Use Committee and performed in accordance with the Animal Care and Use Policies of the University of Wisconsin-Madison M006487-R01-A02. MSC(AD) were isolated from the epididymal fat pad of 10- to 14-week-old mice, as previously published [31]. MSC(BM) were isolated from the femur and tibia of 10- to 14-week-old mice, as previously published [40]. Submandibular MSC(SG) were isolated following the procedure previously published for the isolation of human labial SG MSCs [29]. Mouse MSCs were cultured from tissues derived from at least two mice to limit variability. The cells were treated with their respective cytokines (vehicle, 60 ng/mL IFNγ and/or 10 ng/mL TNFα) for 24 h and labeled with DiI. Each mouse received either allogeneic or syngeneic pooled MSCs. Mouse MSCs were defined by morphology, their ability to differentiate to adipocytes and osteocytes, and the presence of CD44 with the absence of CD45, CD31, and MHCII. Mouse derived MSCs are strongly immunosuppressive, akin to human MSCs, though differences exist in how MSCs by animal source drive immunosuppression [41,42]. Furthermore, similar to human MSCs, mouse MSCs respond robustly to IFNγ pre-licensing [43]. Thus, we considered mouse MSCs a relevant cell to compare to humans for in vivo modeling.

### 2.10 Mouse irradiation and MSC injection

Mice were irradiated in the morning, 24 h prior to MSC injection. Delivery of 15 Gy single dose for the SG irradiation was applied by operating at 220 kV and 13 mA with copper filtration. The dose rate was 2.9032 Gy/min (or 0.048387 Gy/s) [155 s to administer 7.5 Gy per submandibular gland on each side].

MSCs were harvested by trypsinization and labeled (150 K cells/injection to be completed) with Vybrant CM-DiI cell labeling solution (Invitrogen) in accordance with manufacturer's protocol. After labeling the cells, they were resuspended and unilateral injection was completed within 1 h of labeling. A small incision (1–2 cm) was made in the dermis. MSCs were injected unilaterally into the animal's right SG using a 0.5 mL U-100 insulin syringe and a 28G1/2 needle. The cells were injected superficially, viewing the needle tip through the incision.

DiI-labeled MSCs were imaged on an IVIS Spectrum imaging system (PerkinElmer). Mice (n = 3 per treatment group) were anesthetized with isoflurane, as described above, and live images were captured immediately following surgical administration of labeled MSCs, and 1, 6, and 14 days thereafter. Fluorescent signal was monitored using excitation/emission wavelengths of 580/535 nm, respectively. Radiant efficiency was recorded. Images were analyzed using LiveImaging 4.5 software. Additional details are provided in the Supplemental Methods.

### 2.11 Salivary flow measurement

Saliva collection was completed as previously described [44] and as detailed in our Supplemental Methods.

### 2.12 Histological analysis

Mouse submandibular SG tissues were fixed in 10 % formalin (Fisher Chemical), embedded in paraffin and cut into 5-μm sections. H&E staining was completed as described above. The tissues were imaged using an Olympus DP80 microscope and the CellSens software package.

Particle size and thickness of the secretory granular band of the convoluted granular tubules (CGTs) were measured by histological image processing using ImageJ software. Mice treated with radiation demonstrate glandular atrophy [20]. To show the presence of atrophy, we measure the size of SG ducts. Larger or preserved ducts indicate resilience to radiation-induced atrophy. Toward this, the color channels of the original image were separated using the H&E2 deconvolution vector. We applied a basic threshold to the red color channel to segment the secretory granular band. Next, we calculated median secretory granular band thickness using the Local Thickness application. Average granular particle size was determined from the segmentation using a watershed filter and the Analyze Particles application.

Primary unconjugated anti-Aqp5 and anti-CD45 antibodies (Proteintech) antibodies were both diluted 1:100 in 1X PBS + 0.2 % BSA and incubated on the tissues for 2 h at room temperature on separate sections. Negative (no primary antibody) controls were used. Tissues were subsequently washed with 1X TBST. Secondary antibodies included rabbit IgG-AF594.

Fluorescent images were captured using an Olympus BX51 microscope equipped with an Olympus KP70 camera (Olympus America, Inc., Waltham, MA) and all images were captured using a 20× objective. Three optical fields were captured from each of three slides per treatment group. Fluorescent quantification was completed using Fiji Image J2 software. Aqp5 and CD45 MFI were normalized to DAPI MFI for each respective field. Additional details are provided in the Supplemental Methods.

### 2.13 Statistical analysis

All applicable data were analyzed by One-way Anova. Statistical analyses were completed using GraphPad Prism 10 software. Data reported as Avg ± SEM. P < 0.05 was considered statistically significant. RNA sequencing genes were considered differentially expressed for analysis if the adjusted p-value (Benjamini-Hochberg) was <0.05.

## 3 Results

### 3.1 Human MSC(BM), MSC(AD), MSC(SG) transcriptomes are largely similar but have some divergent features

**Fig. 1:**
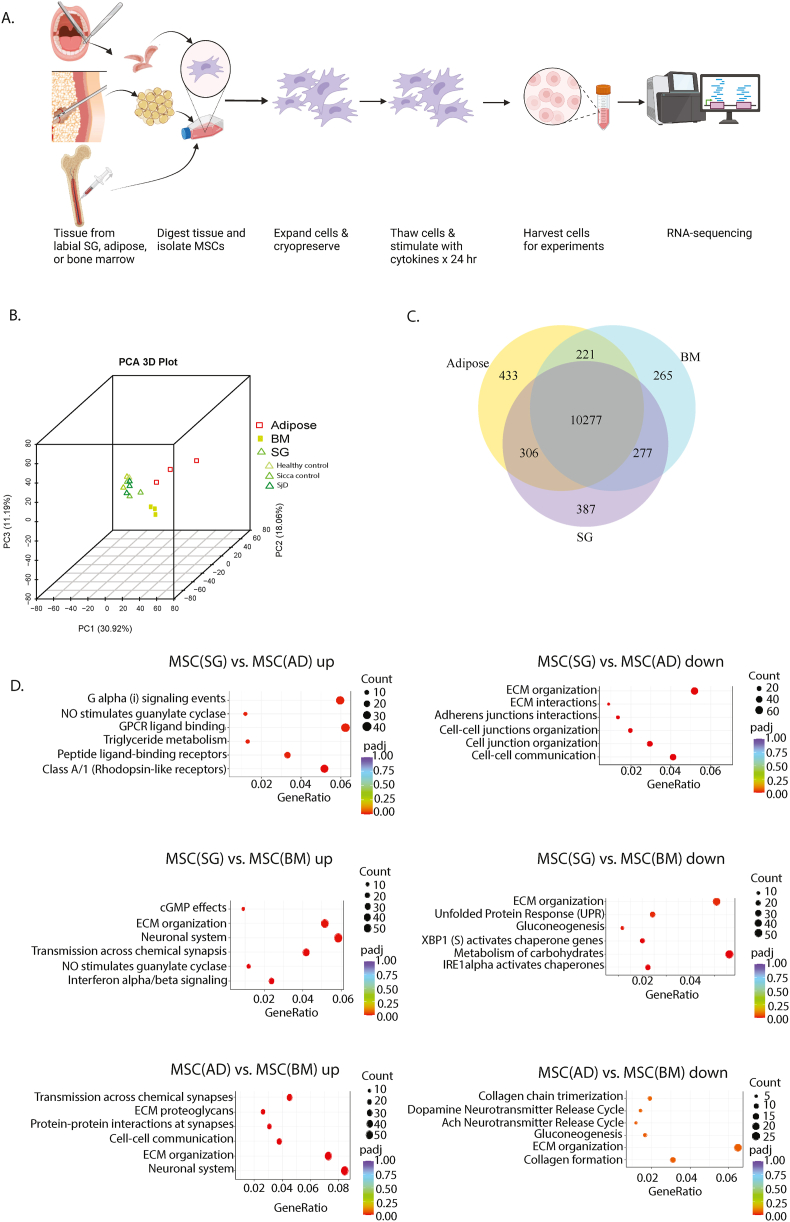
**Human MSC(BM), MSC(AD), MSC(SG) have largely similar transcriptomes but some divergent features**. RNA sequencing was performed on human MSC(SG) (n = 9), MSC(BM) (n = 3), and MSC(AD) n = 3. Three different sources of MSC(SG) were tested: SjD-derived MSC (n = 3), sicca control MSC (n = 3), and healthy control MSC (n = 3). A) Representative figure created in https://BioRender.com; B) PCA of MSCs by source; C) Co-expression Venn Diagram of MSCs by tissue source comparing all MSC(SG) vs. MSC(BM) and MSC(AD); D) Reactome pathways of differentially expressed genes for each comparison. The top six pathways by lowest p-values are shown. Adjusted p-values were calculated with the Benjamini-Hochberg approach.

MSC(BM) and MSC(AD) represent the status quo for MSC translational and therapeutic studies. To compare MSC(SG), we performed bulk RNA-sequencing to understand, in an unbiased manner, how these MSC(SG) are similar and different from the status quo (Fig. 1A) (https://doi.org/10.5061/dryad.9ghx3ffv6). The transcriptome of each untreated MSC type clustered in a distinct manner (Fig. 1B). Regardless of subject status, MSC(SG) tended to cluster together. Most transcripts were shared between MSC sources (85 %), but MSC(AD) had the greatest number of unique transcripts (4 %), followed by MSC(SG) (3 %), and MSC(BM) (2 %) (Fig. 1C). The transcripts most significantly increased in MSC(SG) relative to MSC(AD) were related to CLASSA/1(Rhodopsin-like receptors), a group of G protein-coupled receptors (Fig. 1D; Supplemental Table 2).

MSC(SG)s had fewer transcripts related to cell-cell communication and extracellular matrix. Compared to MSC(BM), MSC(SG) had upregulated pathways related to interferon alpha/beta signaling and neuronal system related transcripts. Top downregulated pathways in MSC(SG) compared to MSC(BM) included IRE1alpha activation of chaperones. These differences persisted when sicca-control and SjD MSCs were excluded from the transcriptomic comparisons (Supplemental Fig. 2). Finally, compared to MSC(BM), MSC(AD) had upregulated transcripts related to the neuronal system (Fig. 1D). There were no significantly enriched downregulated transcripts.

### 3.2 Human MSC(SG) single cell transcriptome differs by cytokine treatment condition

**Fig. 2:**
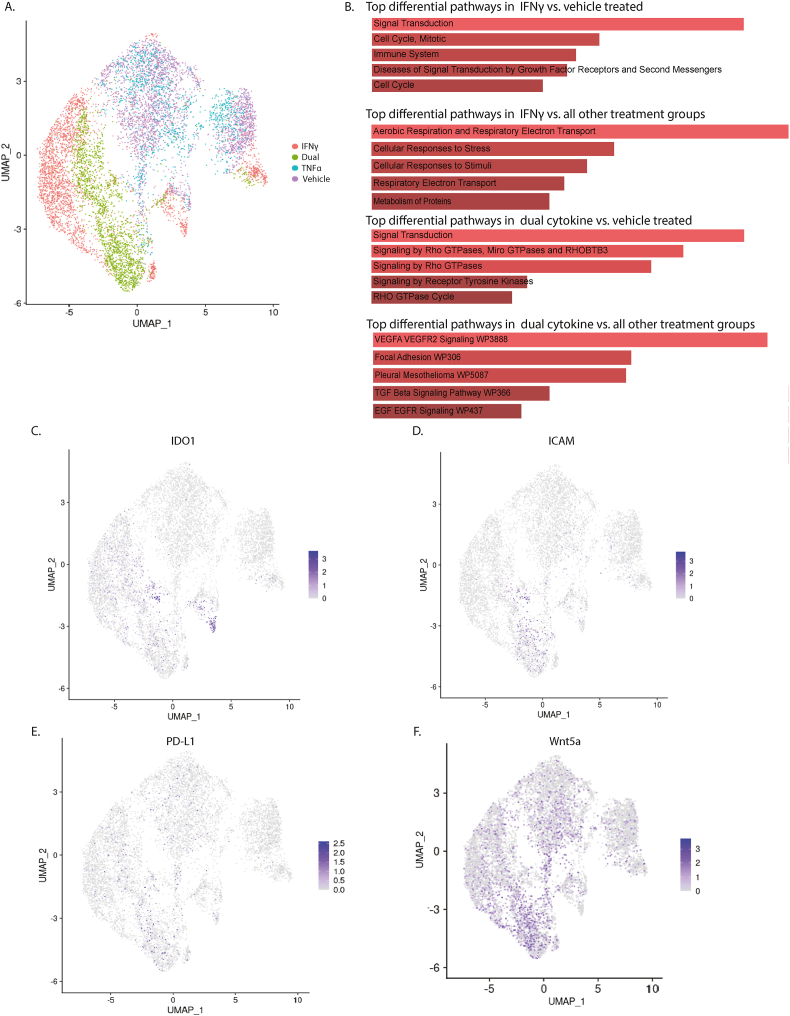
**Human MSC(SG) single cell transcriptome differs by cytokine treatment condition.** Healthy human MSC(SG) (n = 3 biological replicates) were treated with vehicle, 60 ng/mL IFNγ, 10 ng/mL TNFα, or both and cryopreserved for single-cell RNA sequencing. A) UMAP of MSC(SG)s by cytokine treatment condition; B) Top five enriched Reactome pathways of significant transcripts (adjusted p-value ≤0.05 as calculated by the Benjamini-Hochberg procedure) of the represented comparisons; C) UMAP of IDO1 expression; D) UMAP of ICAM-1 expression; E) UMAP of PD-L1 expression; F) UMAP of WNT5 expression.

We performed scRNA seq on MSC(SG) treated with vehicle, IFNγ (60 ng/mL), TNFα (10 ng/mL), or dual cytokine conditions. We found that the vehicle- and TNFα-treated single cell MSC(SG) transcriptomes tended to group together (Fig. 2A); however, IFNγ and dual cytokine conditions had distinct transcriptomic profiles. We compared the top differential transcripts (https://doi.org/10.5061/dryad.9ghx3ffv4) from the IFNγ to vehicle treated MSC(SG)s and identified 928 significant transcripts (Supplemental Table 3). These transcripts represented upregulated pathways related to signal transduction, cell cycle, and the immune system (Fig. 2B). When we compared the top pathways of the IFNγ treated MSC(SG)s to all other treatments, the enriched pathways included aerobic respiration, stress response, electron transport, and metabolism (Supplemental Table 4). In contrast, the dual cytokine treated MSC(SG)s had 2283 significantly differentially expressed transcripts compared to vehicle treated MSC(SG)s (Supplemental Table 5). Their top enriched pathways included signal transduction and signaling through rho GTPases. The most enriched pathways of the dual cytokine treated MSC(SG)s compared to all the other treatment conditions included VEGF signaling, focal adhesion, TGFβ signaling, and epithelial growth factor.

Other researchers find that dual cytokine treatment enhances the immunomodulatory features of MSCs [45]; however, the effect of dual cytokines on MSC(SG) is unknown. We found that dual cytokine treated MSC(SG)s have upregulated transcripts related to immunomodulatory targets including IDO, ICAM-1, and PD-L1 (Fig. 2C–E). Interestingly, our pathway findings above indicated that dual cytokine treated MSC(SG)s had enriched transcript pathways related to epithelial growth and rho GTPases. Because epithelial growth and rho GTPases are related to trophic factors such as Wnts and R-Spondins [46], we evaluated our list of differentially expressed transcripts for these trophic factors. We narrowed our differentially expressed transcripts to 33 transcripts that had a q-value ≤0.05 and a fold change of at least 1.5 in the dual cytokine MSC(SG) compared to vehicle-treated groups. We found the trophic factor, Wnt 5, was increased significantly in the dual cytokine treated MSC(SG)s compared to vehicle treatment conditions (Fig. 2F; Supplemental Table 6).

### 3.3 The trophic secretome differs by MSC source and cytokine stimulation conditions

**Fig. 3:**
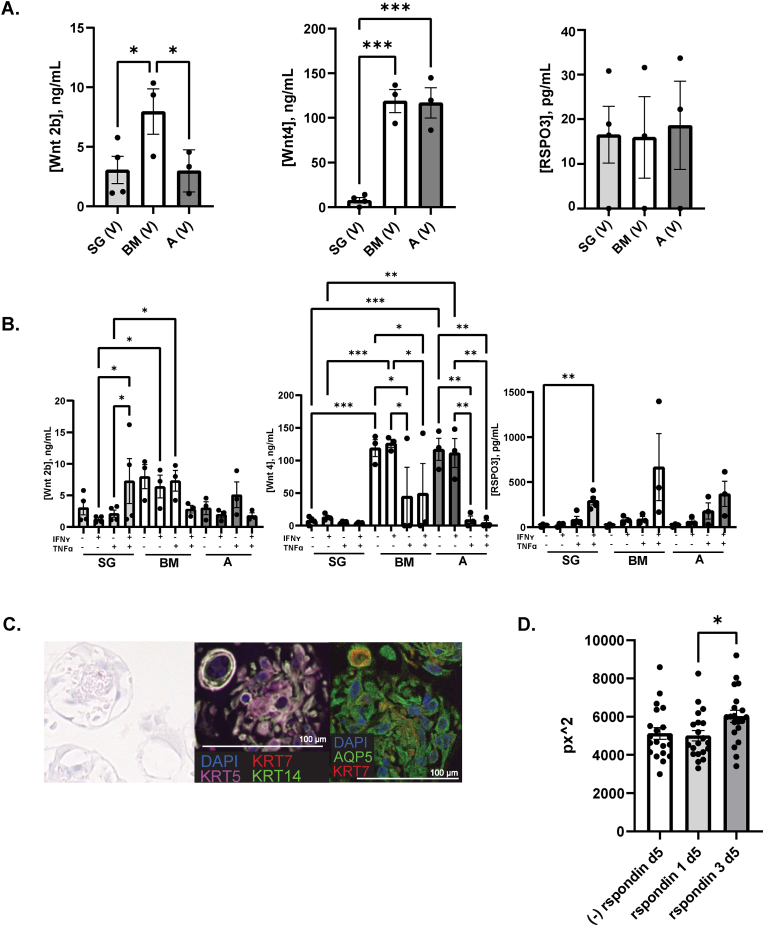
**The trophic secretome differs by MSC tissue source and cytokine stimulation conditions.** Human MSC(BM) (n = 3), MSC(AD) (n = 3), MSC(SG) (n = 4) were cultured with (i) vehicle; (ii) 10 ng/mL IFNγ; (iii) 10 ng/mL TNFα; (iv) 10 ng/mL IFNγ + 10 ng/mL TNFα. Conditioned media were saved for ELISA. A) Secretome proteins from different sources of MSC were compared under vehicle treatment conditions; B) Secretome proteins from different sources of MSC were compared under IFNγ/TNFα treatment conditions; C) Representative figure of organoids displaying differentiation to acinar cells (Aqp5), myoepithelial cells (Krt14), basal ductal cells (Krt5), and luminal ductal cells (Krt7); D) organoids were cultured in medium devoid of R-Spondin or containing, 500 ng/mL RSPO1, or 500 ng/mL RSPO3. Area (pixels squared) of organoids on day 5 of culture conditions. Ordinary ANOVA was used for equal SDs and with unequal SDs, we used Brown Forsythe and Welch ANOVAs. ∗P=<0.05; ∗∗<0.01; ∗∗∗<0.001.

MSCs generate a trophic secretome, capable of supporting local progenitor cell populations [47,48]. We found Wnt5a as a candidate trophic factor on our transcriptomic analysis; however, it is unknown whether cytokine pre-licensing affects this trophic secretome. Thus, we performed an extensive analysis of trophic factors at the protein level. We studied the trophic secretome profile of healthy control untreated culture adapted MSC(SG), MSC(BM), and MSC(AD). Conditioned medium from MSC(BM) had more Wnt2b than MSC(SG) and MSC(AD) (Fig. 3A). MSC(BM) and MSC(AD) produced more Wnt4 than MSC(SG). There was no significant difference between MSC sources in levels of R-Spondin 3 (RSPO3; not shown are results with no significant or measurable protein from Wnt1, GDNF, RSPO1, Wnt3a, and Wnt5a ELISAs). We included additional analyses using MSC(SG) derived from SjD and sicca control subjects. Interestingly, SjD and sicca control subjects had higher levels of Wnt4 than healthy control MSC(SG)s (p < 0.0001 for both comparisons) (Supplemental Fig. 3A–B).

IFNγ and TNFα have well established effects on the immunomodulatory behavior of MSCs [8,49,50]. The effect of these cytokines on the MSC secretome and by MSC source, is unknown. We found that Wnt2b significantly increased in MSC(SG) after treatment with IFNγ (10 ng/mL) and TNFα (10 ng/mL) (Fig. 3B) compared to IFNγ-treated, making Wnt2b levels similar between MSC(SG) and MSC(BM). MSC(BM) seemed to respond less to IFNγ and TNFα than TNFα alone, though this trend was not significant. TNFα reduced Wnt4 in all MSC sources with significance in MSC(BM) and MSC(AD). RSPO3 production was synergistically increased by treatment with both IFNγ and TNFα in all MSCs, regardless of source, though only reaching significance in MSC(SG) (Fig. 3B). Though less common, TGFβ might also modulate MSC function [51,52] so we additionally investigated the effects of TGFβ pre-licensing on the same secretome markers (Supplemental Fig. 3C). Wnt4 was suppressed by TGFβ. TGFβ did not clearly enhance any trophic secretome markers. We did not find clear parallels between transcript and protein level expression.

In colon epithelium, RSPO3 drives stem cell recovery and ultimately promotes regeneration of the epithelium through stem cell differentiation [53]. To determine the impact of RSPO3 on SG stem cell differentiation, we generated SG organoids in standard differentiation medium and either vehicle, RSPO1, or RSPO3. We found that RSPO3 exposure resulted in advanced organoid development over RSPO1 and vehicle treated organoids (Fig. 3C and D).

### 3.4 IFNγ and TNFα combination synergistically enhances MSC(SG) immunomodulatory capacity

**Fig. 4:**
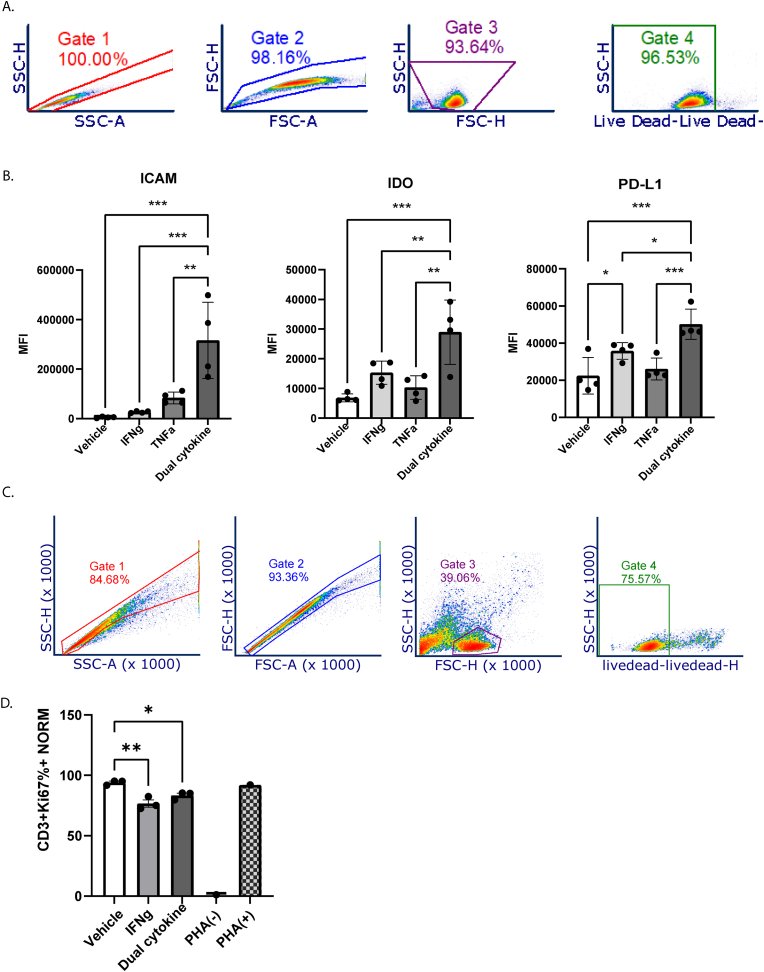
**IFN**γ**/TNFα combination synergistically enhance MSC(SG) immunomodulatory capacity**. Human MSC(SG) (n = 4 healthy controls) were treated with vehicle, 60 ng/mL IFNγ, 10 ng/mL TNFα, or both and cryopreserved. For the described assays, the cryopreserved MSC(SG) were culture rescued for 18 h before proceeding with the described experiments. A) Gating strategy for MSCs; B) Median fluorescence intensity (MFI) of MSC(SG) in each treatment condition (n = 4 biological replicates, each); C) Gating strategy for PBMCs; D) After normalizing for final number of MSCs per well, both cytokine treatment conditions continued to have greater suppression of CD3^+^ T-cell proliferation than vehicle treated MSC(SG). Mean percent positive for Ki67 of total CD3^+^ cells were: vehicle 94 %, IFNγ 77 %, and dual cytokine 83 %. Ordinary ANOVA was used to generate p-values; ∗=<0.05; ∗∗<0.01; ∗∗∗<0.001.

Cryopreservation is critical to the feasibility of using MSCs as therapy; yet cryopreservation impairs MSC immunomodulatory capacity [9,54,55]. IFNγ and TNFα have been studied extensively in other traditional MSC sources, but nothing is known of their effect on MSC(SG) immunomodulatory capacity after cryopreservation. Our scRNA seq data indicated that immunomodulatory markers might increase in MSC(SG) in dual cytokine treatment conditions. We found that after thaw, MSC(SG) pre-licensed with IFNγ (60 ng/mL) and TNFα (10 ng/mL) had higher ICAM-1 than vehicle, IFNγ, or TNFα treated conditions (Fig. 4A and B). We found that IDO and PD-L1 also were highest in the IFNγ and TNFα condition compared to any of the individual treatment conditions. IFNγ alone significantly increased PD-L1 compared to vehicle condition. We did not find a significant difference in immunomodulatory marker expression between different TNFα doses (Supplemental Fig. 4A).

We found that cytokine treated MSC(SG) were superior to vehicle treated MSC(SG) regarding their capacity to suppress T-cell proliferation (Fig. 4C and D; Supplemental Fig. 4B–C). Dual cytokine stimulated MSCs reduced T-cell replication by over 10 % and IFNγ stimulated MSCs by 17 %.

Given our interest in optimizing MSC(SG) as a potential therapeutic, we sought to determine the effect of IFNγ, TNFα, or both on MSC doubling time. We also compared three different enzyme digestion approaches. We saw no difference in doubling time by enzyme digestion approach (Supplemental Fig. 4D). Furthermore, we found no difference in doubling time between MSC(SG) and MSC(BM) and MSC(AD) (Supplemental Fig. 1B).

### 3.5 MSC(SG) pre-licensed with IFNγ and/or TNFα are superior to MSC(SG) without pre-licensing to preserve salivary gland function after radiation in mice

**Fig. 5:**
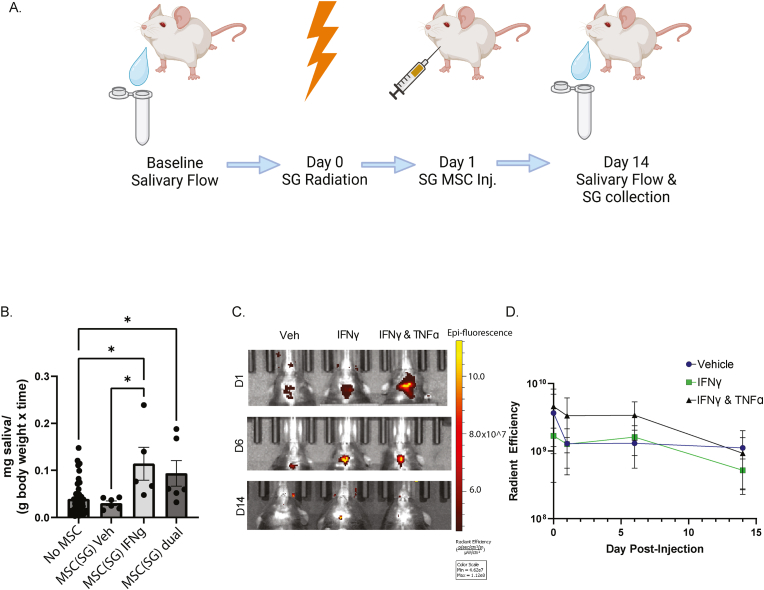
**MSC(SG) pre-licensed with IFNγ and/or TNFα are superior to MSC(SG) without pre-licensing to preserve salivary gland function after radiation in mice.** We measured stimulated salivary flow at baseline. Mouse MSCs were grown from tissues derived from at least two mice and pooled prior to cryopreservation to limit variability. After expanding the cells to 80 % confluence, the cells were treated with their respective cytokine condition for 24 h prior to cryopreservation. MSCs were cryo-recovered for 18 h prior to labeling them with DiI, washing them well, and preparing them for injection. Each mouse received either allogeneic or syngeneic pooled MSCs. On Day 0 the SGs of the mice were irradiated. On Day 1, mice were injected with MSC(SG) pre-licensed with vehicle (n = 6), 60 ng/nl IFNγ (n = 5), or both IFNγ (60 ng/mL) and TNFα (10 ng/mL) (dual cytokine; n = 6). On day 14, stimulated salivary flow was measured and salivary glands were harvested for further analyses. Data points represent individual mice treated with either syngeneic or allogeneic MSCs. A) schematic of treatment plan; B) stimulated salivary flow of mice 14 days after radiation with no MSC treatment (n = 52), on day 14 for mice injected with vehicle-treated MSC(SG) (n = 6), MSC(SG) pre-licensed with IFNγ (n = 5), or MSC(SG) pre-licensed with both IFNγ and TNFα (dual cytokine; n = 6)); C-D) radiant efficiency of injected MSCs at demonstrated timepoints for n = 3 mice in each condition; p-value determined by one-way ANOVA. ∗< 0.05; ∗∗<0.01; ∗∗∗<0.001; ∗∗∗∗<0.0001; RT = radiation therapy.

The effect of MSC(SG) and dual cytokine pre-licensing conditions on preservation of salivary flow after radiation therapy is unknown. We studied the effects of MSC(SG) by cytokine pre-licensing conditions through MSC injection of mice after radiation therapy (Fig. 5A).

We sought to determine if cytokine pre-licensing could optimize the effect of MSC(SG) on preservation of salivary function. We found that, compared to irradiated mice, there was a significant improvement in salivary flow after treatment with MSC(SG) pre-licensed with IFNγ (60 ng/mL) or IFNγ (60 ng/mL) and TNFα (10 ng/mL) (dual cytokine conditions) (Fig. 5B). MSC(SG) without cytokine pre-licensing failed to protect saliva flow.

Although persistence of injected MSCs from other sources is well established, the persistence of locally injected MSC(SG) is unknown. We found that, regardless of cytokine pre-licensing condition, MSC(SG)s persisted at least 14 days in SG tissue (Fig. 5C and D), though persistence seemed to diminish over time.

We found MSC(SG) preservation of salivary flow was improved after dual cytokine treatment, so we compared dual cytokine pre-licensed MSC(SG) to dual cytokine pre-licensed MSCs from other tissue sources. The most robust response to IFNγ and TNFα treatment seemed to result from MSC(SG) treatment but there was a similar trend seen in MSC(AD) (Supplemental Fig. 5A–B). No differences achieved significance, likely due to low numbers in the MSC(BM) and MSC(AD) MSC groups. We also evaluated salivary flow after injection with vehicle-treated MSCs by tissue source. Mice treated with vehicle MSC(SG) lost significantly more salivary flow from baseline compared to vehicle MSC(BM) and MSC(AD) (Supplemental Fig. 5C). We saw no difference in mouse weight with any of our interventions (Supplemental Fig. 5D).

### 3.6 MSC(SG) pre-licensed with both IFNγ and TNFα preserve salivary gland structure and aquaporin 5 compared to vehicle or IFNγ treatment alone

**Fig. 6:**
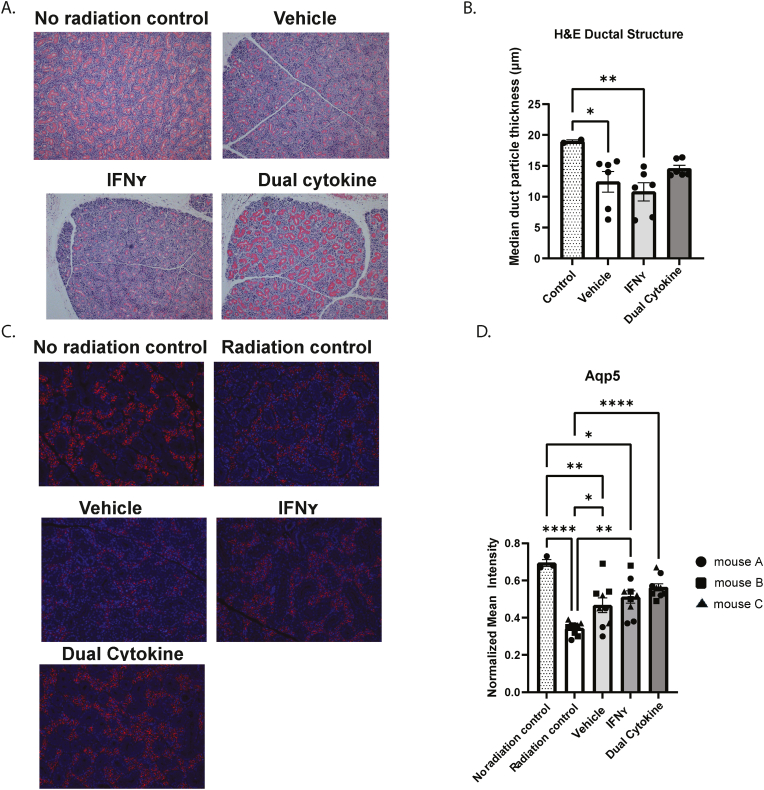
**Structural integrity and Aqp5 are maintained in mouse submandibular salivary glands treated with dual cytokine-stimulated MSCs.** A) Mean particle thickness of ducts of glands injected with MSCs from varying cytokine treatment conditions. A representative image is shown of control mice that did not receive radiation (control; n = 2), MSC-treated mice received MSCs 24 h post radiation that were not pre-licensed (vehicle; n = 6)), pre-licensed with IFNγ (60 ng/mL; n = 6), or dual stimulated with IFNγ (60 ng/mL) and TNFα (10 ng/mL) (dual cytokine; n = 6). Submandibular salivary glands were collected 14 days post treatment and stained for H&E; B) A dot plot showing the duct particle size in each treatment condition; C) A representative image is shown of control mice that did not receive radiation (no radiation control) or received 15 Gy of CT-directed radiation to the submandibular salivary gland (radiation control) or MSC treated mice as defined above and salivary glands were collected 14 days post treatment in all the conditions except radiation controls that were collected at 7 days. FFPE sections were stained for Aqp5 expression. Three optical fields were captured for each slide and we quantified the mean fluorescence of each image (red channel). Data was normalized to DAPI expression (blue channel); D) A dot plot showing the median Aqp5/DAPI for each treatment group. Each animal treated is represented by a different shape (n = 3 mice in each group, except the untreated control [n = 1]). P-value by ANOVA ∗< 0.05; ∗∗<0.01; ∗∗∗<0.001; ∗∗∗∗<0.0001.

We compared the gland structure of non-irradiated mice and irradiated mice treated with MSCs pre-licensed with vehicle, IFNγ (60 ng/mL), or both IFNγ (60 ng/mL) and TNFα (10 ng/mL) (dual cytokine). H&E staining showed preservation of the size of ducts in SG tissue treated with MSC(SG) that were pre-licensed with dual cytokines (Fig. 6A and B). In contrast, IFNγ and TNFα treatment of MSC(BM) and MSC(AD) did not appear to have the same protective effect on duct size as MSC(SG) (Supplemental Fig. 5E). MSC(BM) and MSC(AD) seemed to have the greatest protective effect in the vehicle condition, though small numbers precluded significance. We saw a similar trend when we performed a sensitivity analysis of dual cytokine-stimulated MSC(SG) from allogeneic mice (Supplemental Fig. 5F).

To understand the mechanism driving improved salivary flow with cytokine-stimulated MSCs, we studied aquaporin (Aqp) 5 expression. We analyzed the SGs of the mice at two weeks post-MSC injection, control mice that were not irradiated, and mice that received radiation but no MSC therapy. Immunofluorescence showed preserved amount of Aqp5 after MSC treatment compared to radiation controls (Fig. 6C and D). Dual cytokine treated MSCs reversed the significant Aqp5 loss seen in untreated irradiated mice. To assess the effect of MSCs on the local immune environment, we performed CD45 staining. Radiation significantly reduced CD45 levels compared to untreated mouse SG. MSC treatment conditions demonstrated CD45 expression to levels akin to mice that did not receive radiation (Supplemental Fig. 5G–H).

## 4 Discussion

**Fig. 7:**
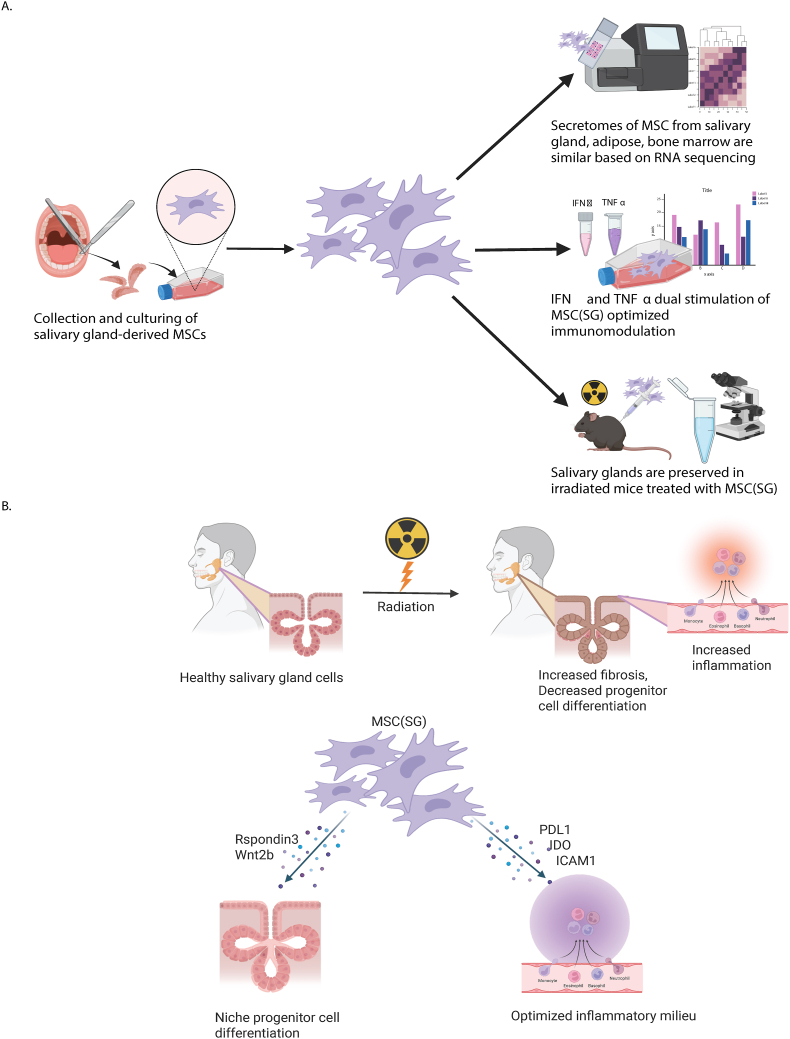
**MSC(SG), particularly after cytokine pre-licensing with IFNγ and TNFα, are a feasible and enhanced MSC source for treatment of radiation-induced xerostomia.** Bulk RNA sequencing of MSC(BM), MSC(AD), and MSC(SG) revealed that they shared 85 % of transcripts. Key differences included extracellular matrix production and response to cytokines in MSC(SG). Regardless of MSC source, dual stimulation of MSCs with IFNγ and TNFα produced an average of more than a 20-fold increase in R-Spondin 3 compared to vehicle conditions. Additionally, IFNγ and TNFα pre-licensing optimized immunomodulatory marker expression more than IFNγ alone. Intercellular adhesion molecule 1 (ICAM-1) increased 12-fold more, programmed death ligand 1 (PD-L1) increased 1.4-fold more, and indoleamine 2,3 dioxygenase (IDO) increased 2-fold more with IFNγ/TNFα pre-licensing than IFNγ alone. Both cytokine stimulation conditions resulted in a 1.2-fold decrease in T-cell proliferation. Gland structure and salivary flow are preserved in irradiated mice treated with MSC(SG) pre-licensed with IFNγ/TNFα. B) proposed mechanism of MSC action. R-Spondin 3 and Wnt2b generated by MSC(SG) promote epithelial regeneration after damage from radiation. Radiation also creates a pro-inflammatory local environment with increased dual positive T cells. MSC(SG) might protect local epithelial through optimizing the local inflammatory milieu like protecting macrophages that are important for tissue repair. McCoy, S. (2025) https://BioRender.com/o01d792. Created in https://BioRender.com.

MSCs are a burgeoning therapeutic option for patients suffering from radiation-induced xerostomia. MSC(BM) and MSC(AD) are currently under study as treatment for radiation induced xerostomia [21,22]. We report on MSC(SG) as an intriguing cell source for treatment of radiation-induced xerostomia. For the first time we show the phenotype and function of MSC(SG) at baseline and with IFN*γ* and/or TNFα stimulation, comparing to MSC(BM), MSC(AD). We showed that IFNγ and TNFα pre-licensed MSC(SG) administered intraglandularly protect saliva production, likely through preserved Aqp5, and glandular tissue architecture after radiation. Although the transcriptomic profile was overall similar between MSCs regardless of source, the trophic secretome differed slightly. In contrast to the effect of source on transcriptomics, dual cytokine pre-licensing with IFNγ and TNFα markedly affects the transcriptome of MSC(SG). Further, dual cytokine treatment increased the production of RSPO3. RSPO3 is a key component of the secretome that allows for intestinal niche progenitor cell repopulation. We showed that RSPO3 optimizes SG organoid growth, supporting that this trophic factor can support stem cell differentiation in SGs. Dual cytokine treatment also increased markers of MSC immunomodulatory activity. We conclude that MSC(SG), particularly after cytokine pre-licensing, might represent an enhanced MSC source for treatment of radiation-induced xerostomia (Fig. 7). We base this conclusion on the fact the MSC(SG) are easy to obtain in a simple outpatient procedure and respond robustly to cytokine stimulation through increased RSPO3 and immunosuppressive properties.

In other organ systems, there is growing recognition that MSCs support local niche progenitor cell replication and differentiation with trophic factors such as Wnts and R-Spondins. R-Spondins amplify canonical Wnt signaling [[56], [57], [58], [59]]. In the intestines, the progenitor crypt cell is Lgr5+. Study of Wnts and R-Spondins in intestinal epithelium progenitor cells showed that Wnts could not induce Lgr5+ progenitor self-renewal alone but rather Wnts were needed to maintain R-Spondin receptors [60]. The presence of R-Spondin receptors drive progenitor cell expansion [60]. RSPO3 production from stromal cells is required for injury repair; mice without RSPO3 have no ability to repair after intestinal injury [53]. Interestingly, we found that under dual stimulatory conditions, there was an increase in RSPO3 in all MSC groups, but significantly in MSC(SG). Further, we found that RSPO3 supported our SG organoid growth. We also found that Wnt2b increased with dual cytokine stimulation. This is salient because Wnt2b is expressed in stromal cells of the submandibular glands during end bud development and is important for morphogenesis [61]. These findings indicate that dual stimulation might optimize key trophic factors to allow recovery of injured local epithelium, though additional mechanistic studies are required to confirm these findings. We found transcription- and protein-level trophic factor responses differed. This could potentially be explained by translational control of the mRNA we measured, a delayed time to protein synthesis, or post-translational modifications. Alternatively, it could be the case that a longer half-life of the trophic factor proteins such as Wnt2b or RSPO3 explain this transcription to protein level expression discordance.

We found that sicca and SjD MSC(SG) had higher levels of Wnt4 than healthy control MSCs. This signifies that MSCs by disease state might have different trophic effects. Further work is required to determine why trophic factors might differ by disease state and the mechanistic impact these changes might have on the local progenitor cell populations.

Post-radiation, SGs have reduced total CD3^+^ cells; however, have increased CD3^+^CD4^+^CD8^+^ subsets [18,62]. In addition to the trophic secretome, we found surrogate markers of MSC immunomodulatory effects, IDO, PD-L1, and ICAM-1 were clearly optimized in the IFNγ and TNFα condition over the IFNγ alone [10,63]; yet, there was no clear difference between IFNγ alone and combined IFNγ and TNFα regarding ability to suppress T-cell proliferation. This might have represented robust T-cell stimulation with PHA, which might have masked subtle differences by cytokine treatment conditions. Nevertheless, cytokine pre-licensing is likely to optimize MSC immunomodulatory effects. Interestingly, we found that CD45 increased with cytokine-stimulated MSC treatment, reaching levels akin to mice that did not receive therapy. It is possible that these levels reflect tissue macrophage restoration, which can promote healing post radiation [[16], [17], [18]]. Alternatively, in other exocrine tissue, epithelial cells can express CD45, which can negatively regulate inflammatory cytokines [64]. Interestingly, CD45 expression in epithelial cells has also been associated with stemness and activation of Wnt pathways [65].

MSCs have been used to treat radiation in mouse models [66]. About half of these studies used intraglandular injections [66]. All used either MSC(BM) or MSC(AD). We describe for the first time the use of MSC(SG). Furthermore, despite the use of pre-licensing in other indications and in human trials, side-by-side comparison of MSC source and cytokine pre-licensing have never been reported. We found that MSC(SG) persist for at least two weeks, which is akin to what has been reported for intraperitoneal or subcutaneous injection persisting at least 10 days [67]. In contrast, MSCs given intravenously persist for three days or less [68]. We did not establish whether the persistence was attributed to live or dead cells, though either state (live or dead) has been hypothesized to convey effect [69,70]. Further, it is not clear whether MSC efficacy is reliant on survival. Indeed, though MSCs are rapidly eliminated after intravenous infusion, the presiding consensus is that MSC apoptosis is a critical method whereby they deploy their immunomodulatory effects [71]. Although the survival of MSCs is not tied to persistence alone, persistence likely contributes to improved efficacy [67]. Further experiments are needed in this use case to determine whether persistence correlates with efficacy.

We found that pre-licensing MSC(SG) with either IFNγ or both IFNγ and TNFα prevented SG dysfunction beyond MSC(SG) treated with vehicle. Given the advantage of dual IFNγ/TNFα therapy on the immunomodulatory and trophic profile of MSC(SG), we proceeded with testing combination therapy among MSCs by source. We did not find a significant difference in saliva flow between vehicle or dual cytokine stimulated MSCs by source; however, these findings are limited by sample size. Additional work with higher sample sizes to account for biological variability is warranted to confirm these results. We showed that MSCs treated with IFNγ and TNFα preserve SG tissue of mice and promote Aqp5 expression. Our data support that MSC(SG) treated with IFNγ and TNFα could serve as a therapeutic tool to treat radiation-induced xerostomia. To transition to clinical use in humans, future work will include research to establish the optimal expansion conditions and dose in human submandibular glands. We do not anticipate difficulties obtaining MSC(SG) as we have successfully obtained these cells from participants with SjD and xerostomia from multiple etiologies [29].

We found that the transcriptional phenotype of MSCs, regardless of the source, might be quite similar, with only a few key differences. MSC(SG) have more G-protein coupled receptors related to chemokine signaling, prostaglandin and endothelin, indicating MSC(SG) may be more sensitive to local inflammation. MSC(SG) might have increased transcripts related to interferon signaling compared to MSC(BM), even when we focused only on MSC(SG) from healthy participants. Past studies of palatine tonsil MSCs, when compared to MSC(BM) and MSC(AD), showed similar results [72]. This finding might reflect the fact that SGs interface with the environment and are a common site of persistent viral infection [[73], [74], [75]]. Compared to MSC(BM), MSC(SG) have fewer transcripts related to IRE-1 alpha chaperone, which is related to the Unfolded Protein Response. MSC aging is promoted by oxidative stress and abnormal Unfolded Protein Response [76]. Further, chronic Unfolded Protein Response stimulation (indicating the unfolded protein response could not be resolved) causes apoptosis [77]. Thus, differential Unfolded Protein Response between MSCs might reflect differential aging and oxidative stress by MSC source. Finally, MSC(AD) and MSC(SG) had more neuronal related transcripts compared to MSC(BM), such as Slitrks, that are responsible for neuron development and growth [22]. This is particularly salient to SG tissue, because salivation is reliant on sympathetic and parasympathetic innervation.

Strengths of our study include that it is the first to compare MSC(SG) to other standard MSC sources used to treat radiation induced xerostomia in mouse models. Furthermore, we report on the effects of cytokine stimulation across these cell types on phenotype and function of the MSCs in the context of radiation induced xerostomia. Despite the strengths of our study, there are several weaknesses. We cannot confirm the baseline demographics of our MSC(BM) and MSC(AD) derived from humans; however, we saw no major differences of any one cell source in each group, indicating there were likely no major aberrant subjects included. We included three biological replicates of MSC(BM) and MSC(AD) in our RNA-seq, and these small numbers could introduce error, and so caution should be used with result interpretation. We found that dual cytokine stimulation markedly increased markers associated with immunomodulatory properties of MSCs including PD-L1, ICAM-1, and IDO. The percentage of actual T-cell suppression was less marked and trended higher in the IFNγ alone than in the dual stimulation group. Though the difference between IFNγ alone and dual cytokine conditions was not statistically significant, the trend could reflect a real finding. If this were the case, it might be explained if IFNγ was responsible for driving inhibition of T-cell replication through mechanisms other than PD-L1, ICAM-1, and IDO such as prostaglandin E2 [78] or nitric oxide [79]. Furthermore, we found an increase in suppression ranging from 10 to 17 % with cytokine stimulation conditions and, though statistically significant, it is unclear whether this level of suppression is clinically significant. Finally, our immunostaining showed increased CD45, which might reflect restoration of tissue macrophages; however, our radiation control glands were harvested 7 days before the other conditions and so our findings could represent natural CD45 repopulation. Additional work to understand the effect of MSC therapy on the local immune environment is warranted. Future work will further elucidate the suppressive effects of MSC(SG) compared to MSC(BM) and MSC(AD). Further studies are needed to determine the optimal MSC dose and timing and to further define the mechanisms driving MSC(SG)'s improvement of dryness and tissue preservation in irradiated mice.

This study provides evidence for the use of dual pre-licensed MSC(SG) for the treatment or prevention of radiation induced xerostomia. Radiation induced xerostomia patients are already being treated with MSC(BM) pre-licensed with IFNγ [80]. The current study supports future clinical trials using dual pre-licensed MSC(SG) based on the ease and cost of procurement, secretome, and immunomodulatory capabilities of these cells.

## Patient and public involvement

Patients and/or the public were not involved in the design, or conduct, or reporting, or dissemination plans of this research.

## Patient consent for publication

Not required.

## Funding

This work was supported by the Specialized Program of Research Excellence (SPORE) program, through the 10.13039/100000054National Cancer Institute (10.13039/100000054NCI) grant P50 CA278595. The work was supported by 10.13039/100000054NCI P30 CA014520, 10.13039/100007015University of Wisconsin Small Animal Imaging & Radiotherapy Facility and by 10.13039/100000072NIDCR R34 DE033042, and by NCI T32 CA009206. The content is solely the responsibility of the authors and does not necessarily represent the official views of the 10.13039/100000002NIH. Support was also received from the Palliative and Supportive Care DOT through the 10.13039/100011089University of Wisconsin Foundation, Ivan and Sandra Haynes Fund.

## Declaration of competing interest

There are no conflicts of interest relevant to this work. Sara McCoy, Jacques Galipeau. ‐‐‐ IFNγ AND TNFα CO-STIMULATION OF MESENCHYMAL STROMAL CELLS DERIVED FROM MINOR SALIVARY (LABIAL) GLANDS FOR THERAPEUTIC USE. PCT patent application 63/548659 filed 2/1/2024.
