# Supplementary material for: IFNγ and TNFα optimize salivary gland mesenchymal stromal cells: an alternative to marrow- and adipose-MSCs for radiation xerostomia
Source: Regen Ther. 2025 Nov 14;30:1086–100. doi: 10.1016/j.reth.2025.11.004 (PMC12663032; doi:10.1016/j.reth.2025.11.004)
Supplement: Multimedia component 1 [file mmc1.pdf]

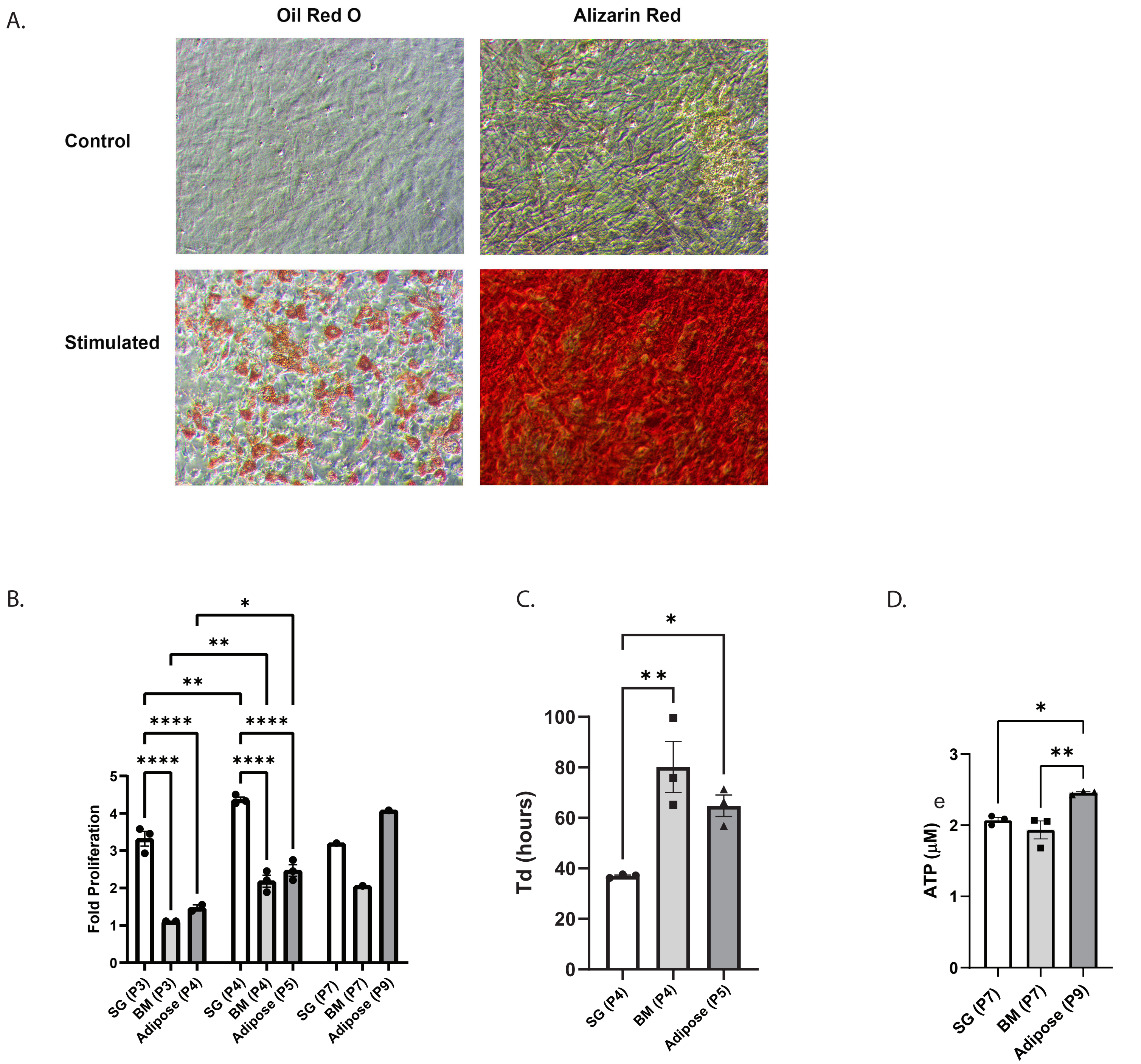

Supplemental Figure 1 MSC differentiation capacity, doubling time, and viability. A) MSCs were placed in differentiation medium. The top left and top right panel are the untreated controls stained with Oil Red O and Alizarin Red, respectively. The bottom left panel MSCs were cultured in adipocyte differentiation medium and stained with Oil Red O. The bottom right panel MSCs were cultured in osteogenic differentiation medium and stained with Alizarin Red; B-C) MSC(SG), MSC(M), and MSC(AD) were plated at passage 3 (P3) or passage 4 (P4). Doubling time was followed over three passages; D) Cell viability for each MSC by source was calculated for MSCs at passage 7 (P7) or passage 9 (P9)
