# Supplementary material for: IFNγ and TNFα optimize salivary gland mesenchymal stromal cells: an alternative to marrow- and adipose-MSCs for radiation xerostomia
Source: Regen Ther. 2025 Nov 14;30:1086–100. doi: 10.1016/j.reth.2025.11.004 (PMC12663032; doi:10.1016/j.reth.2025.11.004)
Supplement: Multimedia component 2 [file mmc2.pdf]

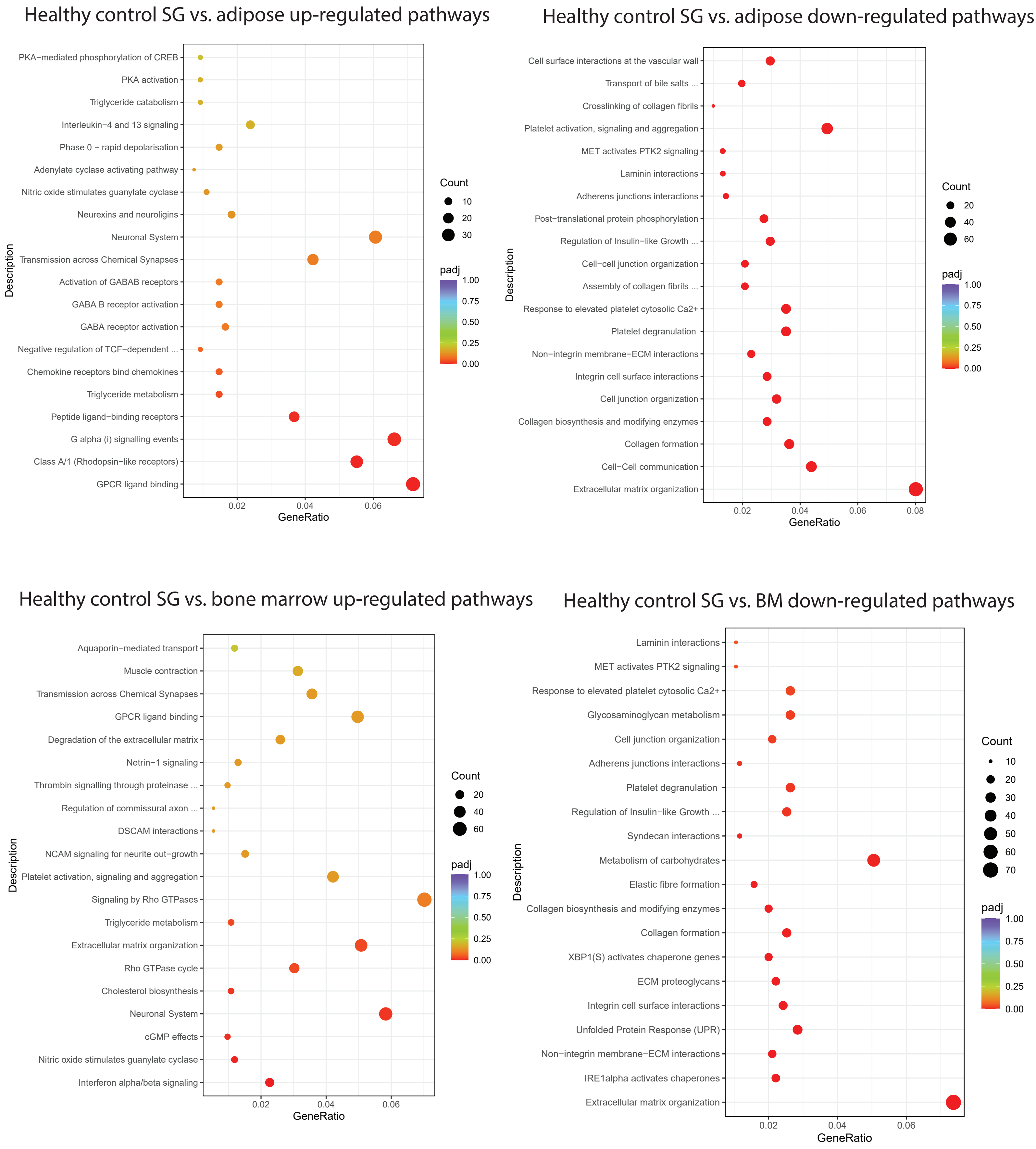

Supplemental Figure 2. Enrichment analyses of MSCs by source. In this figure MSC(SG) include only those derived from healthy controls. n=3 healthy control MSC(SG); n=3 MSC(BM); n=3 MSC(AD). Dot plots show enrichment of reactome pathways in each comparison listed.
