# Supplementary material for: IFNγ and TNFα optimize salivary gland mesenchymal stromal cells: an alternative to marrow- and adipose-MSCs for radiation xerostomia
Source: Regen Ther. 2025 Nov 14;30:1086–100. doi: 10.1016/j.reth.2025.11.004 (PMC12663032; doi:10.1016/j.reth.2025.11.004)
Supplement: Multimedia component 3 [file mmc3.pdf]

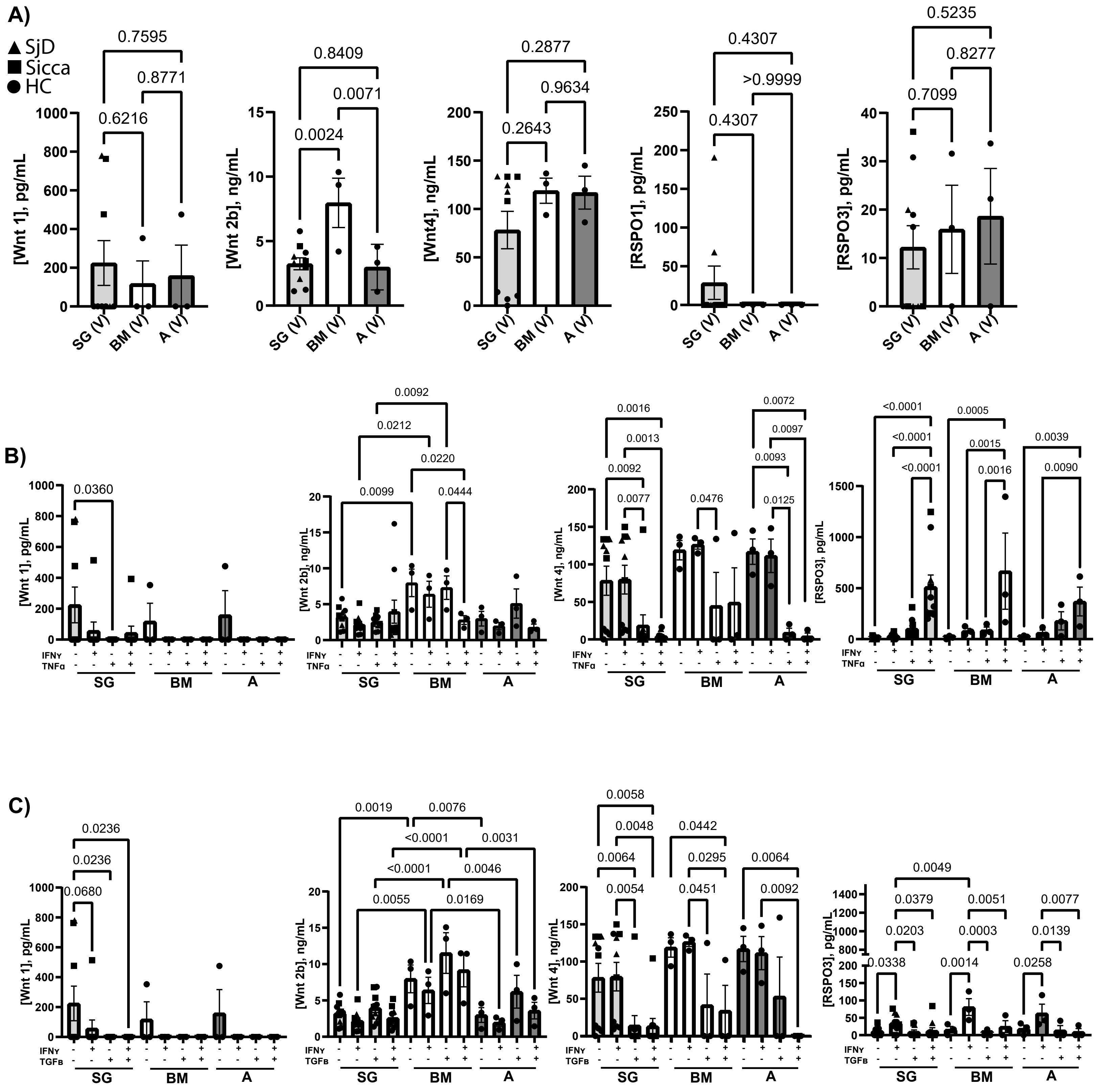

Supplemental Figure 3. The trophic secretome differs by MSC source and cytokine stimulation conditions. MSC(BM) (n=3), MSC(AD) (n=3), MSC(SG) (n=10) were cultured with (i) vehicle; (ii) 10 ng/mL IFNγ; (iii) 2.5 ng/mL TGFβ; (iv) 10 ng/mL TNFα; (v) 10 ng/mL IFNγ + 2.5 ng/mL TGFβ; (vi) 10 ng/mL IFNγ + 10 ng/mL TNFα. In addition to healthy controls, n=3 SjD and n=3 sicca controls were included in the analyses. Conditioned media were saved for ELISA.

A) Secretome proteins from different sources of MSC were compared under vehicle treatment conditions;

B) Secretome proteins from different sources of MSC were compared under IFNγ/TNFα treatment conditions;

C) Secretome proteins from different MSC sources were compared under IFNγ/TGFβ treatment conditions.

Ordinary ANOVA was used for equal SDs.
