# Supplementary material for: IFNγ and TNFα optimize salivary gland mesenchymal stromal cells: an alternative to marrow- and adipose-MSCs for radiation xerostomia
Source: Regen Ther. 2025 Nov 14;30:1086–100. doi: 10.1016/j.reth.2025.11.004 (PMC12663032; doi:10.1016/j.reth.2025.11.004)
Supplement: Multimedia component 4 [file mmc4.pdf]

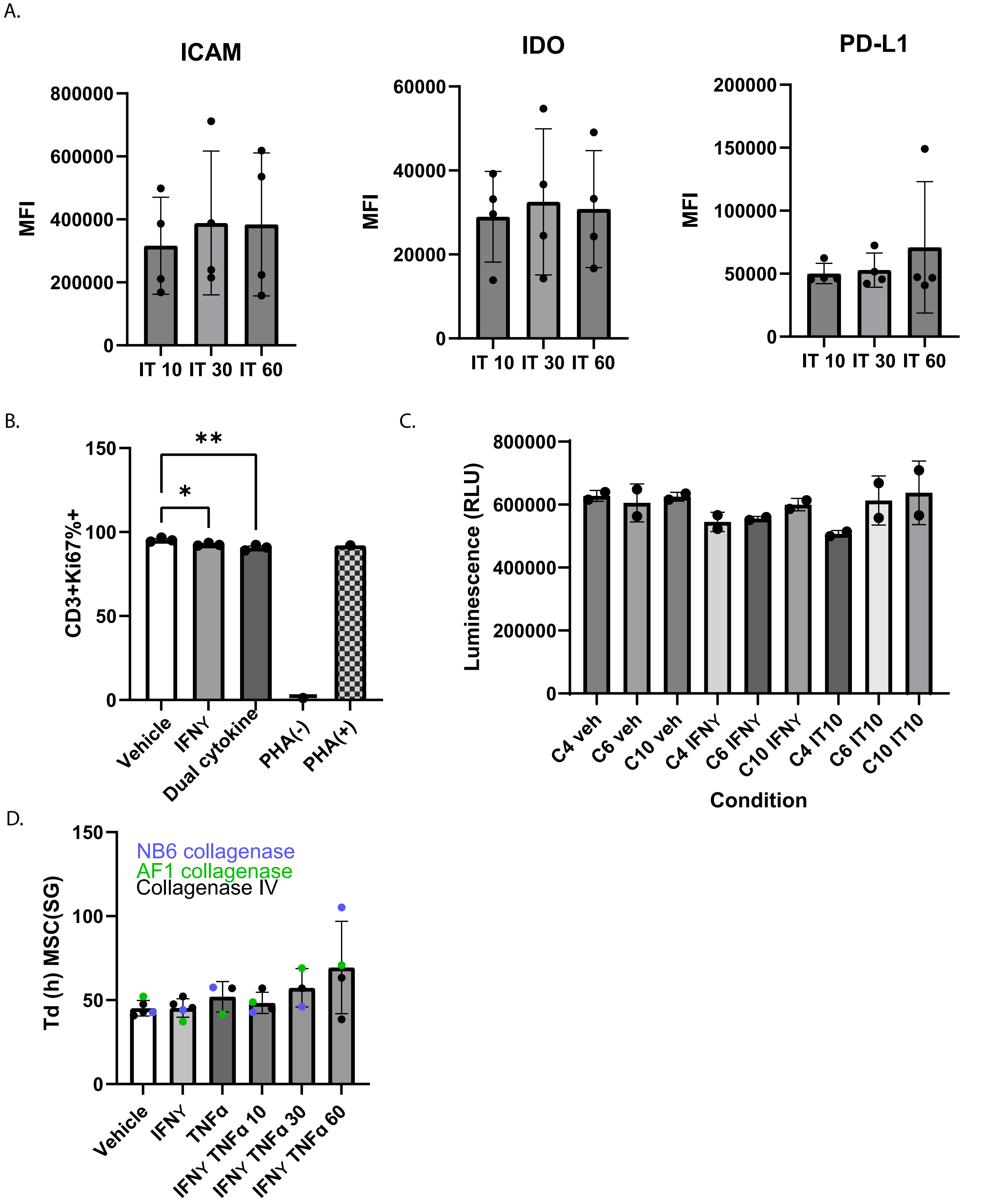

Supplemental Figure 4. The dose of TNFα does not affect immunomodulatory marker expression or doubling time. A) MSC(SG) (n=4) were cultured with (i) 60 ng/mL IFNγ and 10 ng/mL TNFα (IT10); (ii) 60 ng/mL IFNy and 30 ng/mL TNFα (IT30); (iii) 60 ng/mL IFNγ and 60 ng/mL TNFα (IT60). Flow cytometry was performed for the shown markers as in Figure 4. Median fluorescence intensity was reported; B) Both cytokine treatment conditions had greater suppression of CD3+ T-cell proliferation than vehicle treated MSC(SG). Mean percent positive for Ki67 of total CD3+ cells were: vehicle 95%, IFNγ 93%, dual cytokine 91%; C) MSC quantification used to normalize Figure 4D using CellTiter-Glo 2.0 assay for each replicate and condition shown; D) Doubling time of MSCs after cryo-rescue comparing cytokine treatment conditions n=4,each except TNFα and IT30 (n=3, each)
