# Supplementary material for: IFNγ and TNFα optimize salivary gland mesenchymal stromal cells: an alternative to marrow- and adipose-MSCs for radiation xerostomia
Source: Regen Ther. 2025 Nov 14;30:1086–100. doi: 10.1016/j.reth.2025.11.004 (PMC12663032; doi:10.1016/j.reth.2025.11.004)
Supplement: Multimedia component 5 [file mmc5.pdf]

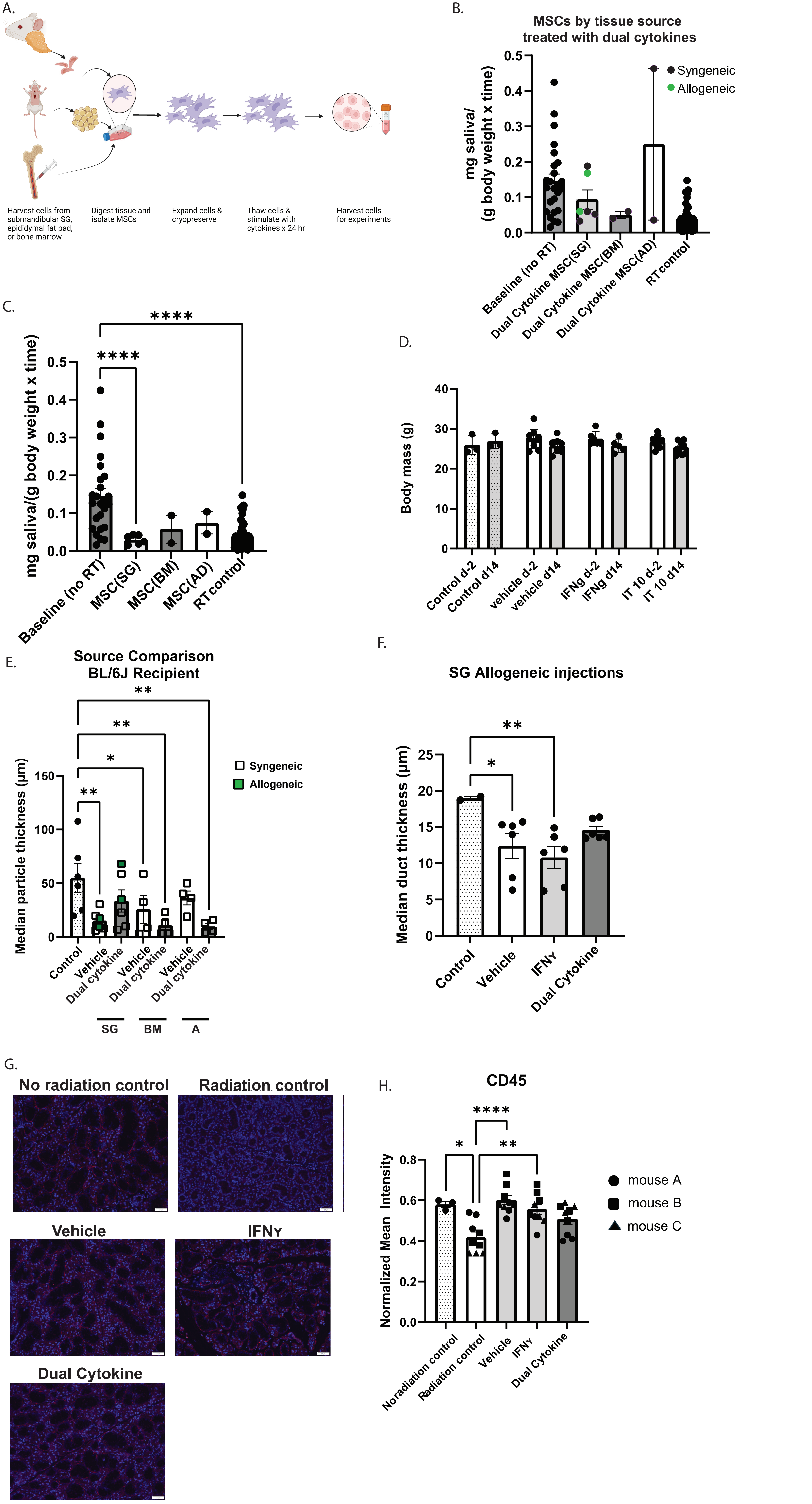

Supplemental Figure 5. Salivary flow and tissue preservation after MSC injection by source and cytokine conditions. We measured stimulated salivary flow at baseline. Mouse MSCs were grown from tissues derived from at least two mice to limit variability. After expanding the cells to 80% confluence, the cells were treated with their respective cytokine condition for 24 hours and cryopreserved. After thaw for 18 hours, the MSCs were injected into the mouse salivary glands. A) MSC workflow schematic; B) stimulated salivary flow normalized for weight and collection time of mice that received MSC(SG), MSC(BM) (n=2), or MSC(AD) (n=2) pre-licensed with dual cytokines, and the radiation (RT) control (n=52); C) stimulated salivary flow at baseline before RT (baseline [no RT]), at day 14 after RT for mice that received MSC(SG) (n=6), MSC(BM) (n=2), or MSC(AD) (n=2) without cytokine pre-licensing, and a control mouse that received radiation but no MSCs (RT control) (n=25); D) Body weights of mice treated in each condition; E) Mean Particle thickness of ducts of gland injected with MSCs from varying tissue sources and cytokine treatment conditions; F) Duct thickness of glands injected with MSC(SG) by cytokine stimulation condition; G) A representative image is shown of control mice that did not receive radiation (no radiation control) or received 15 Gy of CT-directed radiation to the submandibular salivary gland (radiation control) or MSC treated mice as defined above and salivary glands were collected 14 days post treatment in all the conditions except radiation controls that were collected at 7 days. FFPE sections were stained for CD45 expression. Three optical fields were captured for each slide. ImageJ analysis was used to quantify the mean fluorescence of each image (red channel); H) Dot plots showing n=1 mouse for the no radiation control and n=3 mice, each for the other conditions.
