# Supplementary material for: IFNγ and TNFα optimize salivary gland mesenchymal stromal cells: an alternative to marrow- and adipose-MSCs for radiation xerostomia
Source: Regen Ther. 2025 Nov 14;30:1086–100. doi: 10.1016/j.reth.2025.11.004 (PMC12663032; doi:10.1016/j.reth.2025.11.004)
Supplement: Multimedia component 6 [file mmc6.docx]

**SUPPLEMENTAL METHODS**

***Human Samples***

All the work described was performed under the University of Wisconsin Health Sciences IRB 2016-1545, 2018-0815 and 2022-1491 and are completed in accordance with the Declaration of Helsinki. Informed consent was obtained for all human subjects. MSC(AD) were obtained from intra-abdominal adipose tissue and MSC(BM) were sourced from bone marrow as previously described [28] and were derived from deidentified healthy donors. MSC(SG) were obtained from labial salivary gland biopsies as previously described [27, 29]. Epithelial cells were obtained from SGs via explant outgrowth methodology. Briefly, labile salivary gland tissue placed in ice cold 1X HBSS immediately upon excision. The tissue was subsequently washed two times with 1X DPBS. The tissue was minced with a sterile scalpel blade and plated to a Corning T25 flask in a 1:3 mixture of DMEM and F12 media supplemented with 2.5% FBS, 1X Glutamax, 1% penicillin/streptomycin, 10 ng/ml hEGF, 0.4 μg/ml hydrocortisone, and 0.5 μg/ml insulin. The epithelial components were allowed to grow out from the explant pieces to 80% confluence, with media replacement every two to three days. MSC populations were differentially removed by incubation in 0.05% trypsin/0.53 mM EDTA for 3 minutes and further expanded in culture, as previously described [29]. A second 3-minute trypsin/EDTA incubation was completed to enrich the collection of the epithelial cell population.

The epithelial cells were expanded at least one additional time in culture on acidified rat tail collagen 1 (Gibco)-coated plates in KEGM2 (0.06 M Ca^2+^) cell culture media (Promocell) prior to cryopreservation in Cryostore 10 (CS10).

Rat tail collagen I was acidified by dilution with 0.02N acetic acid to a concentration of 50 mg/ml. The collagen I preparation was placed in the tissue culture flask and allowed to incubate for 30 minutes at room temperature. The tissue culture vessel was washed three times with 1X DPBS prior to plating the epithelial cells.

All MSCs met International Society of Cell Therapy including adherence to plastic, differentiation capability (Supplemental Figure 1A), and surface markers [29, 31-33]. Furthermore, all MSCs were evaluated to ensure they had similar doubling time, and they were viable (Supplemental Figure 1B-D). Differentiation assays were performed using R&D Stem X Vivo adipogenic and osteogenic differentiation supplements, in accordance with manufacturer’s protocols.

Demographics were not available for the deidentified MSC(BM) and MSC(AD) healthy donors. Demographics for MSC(SG) are shown in Table 1. Labial SG donors classified as healthy controls volunteered to donate labial salivary glands prior to radiation for head and neck cancer. Labial SGs were not involved in any active disease process. Controls had dry eye and/or mouth symptoms so proceeded to labial SG biopsy, but they did not have any evidence of an autoimmune process driving their symptoms (based on labs and pathology). We termed these patients sicca (meaning symptoms of dryness) controls. Because we were interested in using MSCs to treat other causes of xerostomia in the future, we also collected MSCs from Sjögren’s disease (SjD) subjects met 2016 ACR/EULAR criteria for disease [34].

***Doubling Time and Viability assays***

Pre-licensed MSCs were cryo-recovered from liquid nitrogen storage as described above. The cells were washed and plated (4.2K cells/well) to a 6-well plate. The cells were harvested three days later and counted using a hemocytometer. Doubling time (Td) was calculated: Td = T (h) x ln2/ln (final cell count/initial cell count).

Metabolic ATP production was measured using the CellTiter Glo 2.0 viability assay kit (Promega, Madison, WI). MSC(SG), MSC(BM) and MSC (AD) were seeded to a 96-well plate (1K cells/well). Four days post plating, the media was collected, and the cells were washed and replenished with 100 ml of MSC cell culture media. An equal volume of room temperature CellTiter Glo 2.0 reagent was added to each well. The samples were mixed on an orbital shaker for 2 minutes, with protection from fluorescent lighting. The plate was incubated for 10 minutes at room temperature to equilibrate the signal. Luminescence was recorded using a Biotek Synergy 2 plate reader. Viability was quantified relative to an ATP standard curve spanning from 1 nM to 1 mM concentration (100 ml ATP standard + 100 ml CellTiter Glo 2.0 reagent).

***Cell culture for RNA sequencing***

Sixteen populations of cells (n=3 bone marrow (BM); n=3 omental adipose; n=3 sicca minor salivary gland (MSG), n=3 Sjögren’s MSG and n=4 non-sicca MSG-derived mesenchymal stromal cells) were grown in base media with six different cytokine conditions. The base media was composed of Dulbecco’s modified Eagle’s medium (DMEM, cat. # 10-017-CV, Corning, Corning, NY) + 1% pen/strep (cat. # 30-002-CI, Corning, Corning, NY) +1% L-glutamine (cat. # 25-005-CI, Corning, Corning, NY) + 10% human platelet lysate (hPL, cat. # PLTMax100R, Millcreek Life Sciences, Rochester, MN) + 2 units/mL of heparin. Cells were seeded at such density as was necessary for their successful growth (~3-5K/cm^2^ for BM, ~7-10K/cm^2^ for adipose and ~1-4K/cm^2^ for MSG-derived MSCs) and grown for ~2-8 days in 10-cm plates with 10 mL of media, with media changes performed every other day until the confluence was estimated to be ~60-80%. At that point, the media was withdrawn, the cells were washed once with 7.5 mL of PBS and then 10 mL of serum-starving media was added (base media with 1% hPL, instead of 10%). After 24 hours, the serum-starving media was withdrawn and 10 mL of media in one of six cytokine conditions was added: (i) no cytokines (two plates); (ii) 10 ng/mL IFNγ (cat. # 300-02, PeproTech, Cranbury, NJ); (iii) 2.5 ng/mL TGFβ (cat. # 100-21, PeproTech, Cranbury, NJ); (iv) 10 ng/mL TNFα (cat. # 570102, BioLegend, San Diego, CA); (v) 10 ng/mL IFNγ + 2.5 ng/mL TGFβ; (vi) 10 ng/mL IFNγ + 10 ng/mL TNFα – in each instance, with D10hPL media as the base. All plates were held for 48 hours, with bright-field images of a representative field documented at the very end of that time frame. All plates were cultured at 37° C and 5% CO_2_.

At time of harvest, all steps were performed on ice: the conditioned media (CM) was withdrawn and saved. Specifically, the conditioned media was spun at 4,000 rpm, for 10 min at 4° C in order to remove any stray cells. After the spin, the CM was decanted into a fresh tube. Two aliquots, 1 mL each, were set up in microcentrifuge tubes. All the samples at -80° C until further aliquoting and use in ELISA assays.

Keeping the plates on ice, each plate was washed with 7.5 mL PBS, and the PBS was aspirated; then the plate was tilted to almost 90° to the surface, to let residual buffer collect in one spot, and it was aspirated up. At that point, 600 µL of cold lysis buffer (from CST#9803 10x lysis buffer; but supplemented with 1 mM each of EDTA, vanadate, PMSF and sodium fluoride) was added; the plate was tilted to spread this; it was then scraped, going across the whole surface about 3-5 times, tilted to collect the fluid using the cell scraper in one little spot, and finally the lysate was collected into a cold labeled microcentrifuge tube on ice. These lysates were frozen at -80° C. Bright-field microscopy was used to ensure that the plates were devoid of cells after scraping is done. In the case of the ‘Veh RNA plate’, the only difference was that 1.25 mL of Zymo Research Tri Reagent (cat. # R2050, ZymoResearch, Irvine, CA) was added in place of 600 µL of lysis buffer.

For RNA-Seq studies, 625 µL of RNA in TriReagent was used as a starting point for RNA isolation and purification using the DirectZol RNA Miniprep Plus Kit (cat. # R2070, ZymoResearch, Irvine, CA) according to the manufacturer’s instructions, with elution into 50 µL of provided ultrapure water. After assessment of RNA purity and concentration via absorbance at 200-300 nm by Nanodrop, the isolated RNA was provided to Novogene, Inc (Sacramento, CA) for sequencing to determine genes differentially transcribed between the different MSC sources.

***ELISA***

As for ELISA studies, conditioned media was thawed, pipetted/diluted with manufacturer’s recommended dilution buffer into PCR strip tubes before being dispensed from there in duplicate via multi-channel pipette into the columns of 96-well plates of ELISA kits, with wells pre-coated with capture antibodies for each analyte of interest. For example, a dilution factor of 2:3 refers to 150 µL of conditioned media plus 75 µL of diluent, and a 1:5 dilution factor refers to 45 µL of conditioned media plus 180 µL of diluent. Concentrations of the analytes in the original conditioned media were determined by using 4-parameter logistic regression in GraphPad Prism with interpolation based on the absorbance of the standards in the kits.

MSCs were derived from human bone marrow [MSC(M)] (n=3), omental adipose tissue [MSC(AT)] (n=3), and labial salivary glands [MSC(SG)] (n=9). MSCs were isolated, expanded, and frozen from each tissue source, using three biological replicates for our experiments. Thawed MSCs from each tissue source were cultured to 80% confluence in standard culture media with platelet lysate supplement for all MSCs. MSCs were treated with IFNγ (10ng/mL), TNFα (10ng/mL), or TGFβ (2.5ng/mL). After 48 hrs of culture, the conditioned media was used for ELISA of epithelial morphogens Wnt2b and R-spondin3 (RSPO3).

| analyte | Vendor | Cat. # | dilution factors | | | | | |
| --- | --- | --- | --- | --- | --- | --- | --- | --- |
|  |  |  | Veh | IFNγ | TGFβ | TNFα | IFNγ + TGFβ | IFNγ + TNFα |
| GDNF | Innovative Research (Novi, MI) | IHUGDNFKT | 2:3 | 2:3 | 2:3 | 2:3 | 2:3 | 2:3 |
| RSPO1 | CUSABIO (Houston, TX) | CSB-EL020550HU | 1:1 | 1:1 | 1:1 | 1:1 | 1:1 | 1:1 |
| RSPO3 | Innovative Research (Novi, MI) | IHURSPO3KT | 2:3 | 2:3 | 2:3 | 2:3 | 2:3 | 2:3 |
| Wnt1 | CUSABIO (Houston, TX) | CSB-EL026128HU | 1:1 | 1:1 | 1:1 | 1:1 | 1:1 | 1:1 |
| Wnt2b | CUSABIO (Houston, TX) | CSB-EL026134HU | 1:5 | 1:7 | 1:7 | 1:4 | 1:7 | 1:4 |
| Wnt3a | CUSABIO (Houston, TX) | CSB-EL026136HU | 1:1 | 1:1 | 1:1 | 1:1 | 1:1 | 1:1 |
| Wnt4 | Raybiotech (Norcross, GA) | ELH-WNT4 | 1:8 | 1:8 | 1:10 | 2:3 | 1:10 | 2:3 |
| Wnt5a | CUSABIO (Houston, TX) | CSB-EL026138HU | 1:1 | 1:1 | 1:1 | 1:1 | 1:1 | 1:1 |

***Organoid generation and quantification***

Epithelial cells were cryo-recovered by culture on an acidified rat tail collagen 1-coated flask in KEGM2 media as described above. The epithelia were expanded to 80% confluence. Organoid culture media was prepared by supplementing DMEM (40%)/F12 (60%) with 1% Glutamax, 1% N2, 20 ng/ml EGF, 20 ng/ml FGF-2, 10 μg/ml human insulin, 1 μM dexamethasone, 10 μM Y-27632, 100 ng/ml Wnt3a, 12.5 ng/ml noggin, 1% penicillin/streptomycin [40, 41]. Matrigel (Corning, 354248) was diluted to a concentration of 10 mg/ml with the organoid culture media. The epithelial cells (25K) were resuspended in 50 μl of the diluted Matrigel. Matrigel domes (10 μl) were plated to the center of a well of a 96-well plate (5K cells per 10 μl Matrigel). Duplicate wells were plated for each treatment. Organoids were cultured for five days in the organoid culture media either without supplementation or supplemented with 500 ng/ml R-Spondin 1 or 500 ng/ml R-Spondin 3. Media was replaced every two days.

R-Spondin 1 and 3-treated organoid cultures were imaged daily using an Olympus CK40 light microscope, selecting an optical field in each Matrigel dome that contained at least 10 organoids. Q-path analysis of individual organoid area was calculated for 10 organoids per image. The data represents the average area (p^2) (± SEM) for the 20 organoids per treatment resultant from two individual wells.

Immunohistochemistry (IHC) and immunofluorescence (IFC) analysis was completed on organoids cultured in the organoid culture media described above that was supplemented with R-Spondin 1. The organoids were harvested with Corning Cell Recovery solution in accordance to manufacturer’s protocol. The organoids were subsequently resuspended in 2% agarose [42], embedded in paraffin and sectioned. Tissue sections were baked at 60C for 20 min. Paraffin was cleared with xylene and the tissues were washed with 100% EtOH and rehydrated. H&E staining was completed by dipping the slides in Harris hematoxylin solution (Poly Scientific R&D Corp) for 45 seconds and eosin Y solution (1% alcoholic, G Biosciences) for 30 seconds, which was followed by dehydration and coverslipping using Cytoseal XYL (Epredia).

Immunofluorescence staining was completed on the baked, deparafinnized, and rehydrated tissues as described above. Antigen retrieval was completed in 10 mM Citrate pH 6.0 buffer, using a Biocare Medical (Pacheco, CA) decloaking chamber, according to manufacturer’s protocol. Slides were washed with 1X TBST and blocked for 1 hour in 2.5% (w.v) normal horse serum (Vector Labs). Fluorochrome-conjugated antibodies for Keratin 5 (Krt5-AF647) (1:100, Abam), Keratin 7 (Krt7-AF594) (1:250, Biolegend), and Keratin 14 (Krt14-FITC) (1:100, EMD Millipore) and Aquaporin 5 (Aqp5-AF647) (1:500, Santa Cruz Biotechnology) were diluted in 1X PBS, applied to the tissue and incubated overnight at 4C (Supplemental Table 1). Coverslipping was completed using Vectashield + Dapi (Vector Laboratories) according to manufacturer’s instructions, sealing the slides with clear nail polish.

Fluorescent imaging of the organoids was conducted at the UW–Madison Optical Imaging Core using a Nikon AXR Confocal Microscope, with excitation provided by 405 nm, 561 nm, and 640 nm lasers*.*

***Flow Cytometry***

Pre-licensed MSCs were cryo-recovered from liquid nitrogen storage by slowly resuspending the thawing cells in a-MEM media supplemented with 10% charcoal-stripped FBS, 1% Glutamax and 1% penicillin/streptomycin. The cells were cultured for 18 hours prior to harvest by trypsinization (0.05% trypsin/0.53 mM EDTA, Corning). MSCs (200K/per sample) were stained with the following fluorochrome-conjugated antibodies: Indoleamine 2,3-dioxygenase (IDO)-FITC (1:100, Invitrogen), CD54 (ICAM-1)-APC (1:100, Miltenyi Biotec) and CD274 (PD-L1)-BV421 (1:100, BD Biosciences). Intracellular staining of IDO was completed using the eBiosciences intracellular fixation and permeabilization buffer set, in accordance with manufacturer’s instructions. The PBMCs were stained with Ki67-PE (1:100, BD Biosciences) and CD3-FITC (1:100, BD Biosciences). Intracellular Ki67 staining was completed using the eBiosciences FoxP3 fixation/permeabilization kit, in accordance with the manufacturer’s protocol. Live/Dead cell populations were assessed using Ghost Red 780 viability dye (Tonbo Biosciences). Flow cytometry was performed using an Attune NxT flow cytometer (Thermo Fisher Scientific) and analyzed using FCS Express flow cytometry software.

***Mouse MSC isolation and culture***

All animal experiments were approved by the University of Wisconsin-Madison Institutional Animal Care and Use Committee and performed in accordance with the Animal Care and Use Policies of the University of Wisconsin-Madison M006487-R01-A02. Male Balb/c and C57BL/6J mice were used for these studies. Mice were maintained in a 12-hour light/dark cycle and fed Teklad Global 2018 rodent diet (Inotiv), ad libitum. Mice were euthanized by CO_2_ asphyxiation. Tissues were dissected and placed in cold HBSS until primary cell isolation/culture.

MSC(AD) were isolated from the epididymal fat pad of 10- to 14-week-old mice, as previously published [32]. Tissues were minced and digested in 2.0 mg/ml collagen IV (Worthington) for 20 minutes at 37C with gentle rotation. The filtered cells were washed and plated to a Falcon T-75 flask containing a-MEM media supplemented with 20 % charcoal-stripped FBS, 12.5 mM L-Gln and 1% penicillin/streptomycin. MSC(BM) were isolated from the femur and tibia of 10- to 14-week-old mice, as previously published [43]. The extruded BM preparation was filtered and plated to a Corning T-25 flask containing a-MEM supplemented with 20% charcoal-stripped FBS, 12.5 mM L-Gln and 1% penicillin/streptomycin. Submandibular MSC(SG) were isolated following the procedure previously published for the isolation of human labial salivary gland MSCs [29]. The tissue was digested for 40 minutes at 37C, with gentle rotation. The filtered tissue was plated to a Corning T-25 flask containing DMEM media supplemented with 20% charcoal-stripped FBS, 2.5 mM Glutamax, and 1% penicillin/streptomycin. Mouse MSCs were grown from tissues derived from at least two mice to limit variability. After expanding the cells to 80% confluence, the cells were treated with their respective cytokine condition for 24 hours, labeling them with DiI, washing them well, and preparing them for injection. Each mouse received either allogeneic or syngeneic pooled MSCs. Mouse MSCs were defined by morphology, their ability to differentiate to adipocytes and osteocytes, and the presence of CD44 with the absence of CD45, CD31, and MHCII. Mouse derived MSCs are strongly immunosuppressive, akin to human MSCs, though differences exist in how MSCs by animal source drive immunosuppression [44, 45]. Furthermore, similar to human MSCs, mouse MSCs respond robustly to IFNγ pre-licensing [46]. Thus, we considered mouse MSCs a relevant cell to compare to humans for in vivo modeling.

***Mouse irradiation and MSC injection***

Mice were irradiated 24 hours prior to MSC injection using an Xstrahl Small Animal Radiation Research Platform (SARRP, Xstrahl, UK). Mice were anesthetized in a chamber with 3-5% isoflurane at 1-2 L/min O_2_. Mice were then moved to a bed fitted with a nose cone, bite bar and head cradle within the SARRP and maintained with 1-3% isoflurane for the duration of treatment. Animals were placed on a bed in the ventral recumbent (prone) position with the forelimbs tucked underneath the body to prevent any unintentionally absorbed dose in the paws. Using the MuriPlan software, a Cone-Beam CT image was acquired with the X-ray tube operating at 60 kV and 0.8 mA with aluminum filtration and a protocol was established for administering a total of 15 Gy split between two beams at 90 and -90 degrees to the animals’ neck to affect both sides equally. A 10x10 mm fixed collimator was used to target as much of the salivary glands as possible, while sparing other tissues in the region. By using two lateral beams rather than a single vertical beam at 0 degrees, the intent was to direct more energy to the tissue of interest. Delivery of 15 Gy single dose for the salivary gland irradiation was applied by operating at 220 kV and 13 mA with copper filtration. The dose rate was 2.9032 Gy/min. (or 0.048387 Gy/sec) [155 seconds to administer 7.5 Gy per submandibular gland on each side].

Pre-licensed, cryopreserved MSCs were cryo-recovered for 18 hours, as described above. MSCs were harvested by trypsinization and labeled (150K cells/injection to be completed) with Vybrant CM-DiI cell labeling solution (Invitrogen) in accordance with manufacturer’s protocol. After labeling the cells and washing, the cells were resuspended in a total volume of 30 μl of PBS and maintained in the dark until injection. Unilateral injection was completed within 1 hour of labeling.

In preparation for MSC injection, mice received an IP injection of meloxicam (Norbrook, 10 mg/kg) prior to surgery. The animals were anesthetized with isoflurane isoflurane (3% isoflurane at 1-2 L/min O_2_) throughout the procedure. The cervical/neck area was shaved and prepped for surgery with betadine and 70% EtOH. A small incision (1-2 cm) was made in the dermis, mid-line above the submandibular salivary glands. MSCs were injected unilaterally into the animal’s right salivary gland using a 0.5 ml U-100 insulin syringe and a 28G1/2 needle. The cells were injected superficially, viewing the needle tip through the incision. The skin incision was closed using Vetbond surgical glue (3M). All animals were monitored for pain and discomfort for 48 hours, post-surgery.

DiI-labeled MSCs were imaged on an IVIS Spectrum imaging system (Perkin Elmer). Mice (n=3 per treatment group) were anesthetized with isoflurane, as described above, and live images were captured immediately following surgical administration of labeled MSCs, and 1, 6, and 14 days thereafter. Fluorescent signal was monitored using excitation/emission wavelengths of 580/535 nm, respectively. Radiant efficiency was recorded. Images were analyzed using LiveImaging 4.5 software.

***Salivary flow measurement***

Mice were placed in a clean cage and fasted for two hours prior to the initiation of the saliva collection. Saliva collection was completed as previously described [46]. Mice were anesthetized with isoflurane, as described above, and injected, IP, with 1 mg/kg pilocarpine HCl (Millipore Sigma). Saliva collection was initiated exactly 4 minutes after pilocarpine administration to pre-weighed collection tubes. Collection continued for 15 minutes. Saliva was absorbed with Salivabio children’s swabs (Salimetrics). Following collection, the swab/collection tube apparatus was weighed and data recorded as mg saliva collected/(body weight) x (min). Salivary flow was measured prior to radiation treatment and on day 14 post-surgery.

***Histological Analysis***

Mouse submandibular salivary gland tissues were fixed in 10% formalin (Fisher Chemical), embedded in paraffin and cut into 5-micron sections. H&E staining was completed as described above. The tissues were imaged using an Olympus DP80 microscope and the CellSens software package.

Particle size and thickness of the secretory granular band of the convoluted granular tubules (CGTs) were measured by histological image processing using ImageJ software. Mice treated with radiation demonstrate glandular atrophy [20]. To show the presence of atrophy, we measure the size of salivary gland ducts. Larger or preserved ducts indicate resilience to radiation-induced atrophy. Toward this, the color channels of the original image were separated using the H&E2 deconvolution vector. We applied a basic threshold to the pink color channel to segment the secretory granular band. Next, we calculated median secretory granular band thickness using the Local Thickness application. Average granular particle size was determined from the segmentation using a watershed filter and the Analyze Particles application.

Aqp5 and CD45 IFC was completed on formalin-fixed, paraffin-embedded tissues that were baked, deparaffinized and rehydrated as described above for the organoid IFC. Antigen retrieval was completed, using either 10 mM Citrate pH 6.0 (Aqp5) or 10 mM Tris, 1 mM EDTA, pH 9.0 (CD45) buffer. Slides were washed with 1X TBST and blocked for 9 min in 1% (w.v) Casein (Fisher Scientific). Primary unconjugated anti-Aqp5 and anti-CD45 antibodies (Proteintech) antibodies were both diluted 1:100 in 1X PBS + 0.2% BSA and incubated on the tissues for 2 hours at room temperature on separate sections. Negative (no primary antibody) controls were incubated with 1X PBS + 0.2% BSA. Tissues were subsequently washed with 1X TBST. The secondary antibody (anti-IgG, Invitrogen), rabbit IgG-AF594, was diluted 1:100 (Aqp5) or 1:500 (CD45) in the 1X PBS + 0.2% BSA diluent prior to incubation on the slides for 30 minutes at room temperature. The tissues were washed, and cover slips were applied using Prolong Diamond Anti-fade + Dapi mounting reagent (Invitrogen).

Fluorescent images were captured using an Olympus BX51 microscope equipped with an Olympus KP70 camera (Olympus America, Inc., Waltham, MA) and an X-cite Mini + white-light LED light source (Excelitas Technologies). Imaging was performed using a DAPI-5060C-OFF and TxRed-4040C-OFF filter sets (Semrock Brightline). All images were captured using a 20X objective. Three optical fields were captured from each of three slides per treatment group.

***Collagenase Digestion and Comparison***

In order to establish an optimized protocol for collagenase digestion of our labial salivary glands and establish viability regardless of digestion approach, we compared three commercially available collagens. This included Collagenase IV (Worthington, Waltham, MA), NB6 (Nordmark Pharma), and AF1 (Nordmark Pharma). The latter of the two come are Good Manufacturing Practices-compliant and so can feasibly be used for production of human therapeutic cellular products.

We first prepared our digestion enzymes:

- Collagenase IV- Place 33 mg collagenase IV in a 50-ml conical cf tube. Add 11 mls 1X DPBS. Filter sterilize through a 0.2 micron syringe filter.
- Collagenase NB6 and Hyalenex- Add 1 ml HBSS to the vial (Stock, 100 mg/vial; 10 U/vial) of Collagenase NB6 (located in the cold cabinet) to generate a 100 mg/ml stock solution. Keep on ice. Freeze aliquots at -20 C.
- Collagenase AF-1: Add 1 ml Lactated Ringers to the vial of Collagenase AF-1 (located in the cold cabinet). Neutral protease: Add 1 ml Lactated Ringers to the vial of Neutral Protease (located in the cold cabinet).

To prepare the tissues, we washed the minor salivary glands twice with 1 mL 1x DPBS. We transferred the washed glands to the side of a 50 mL conical tube and the tissue was minced with a scalpel into 1-2mm pieces. We added 3.0 mg/ml Collagenase IV, 0.4 pzu/ml Collagenase AF1 or 0.5 pzu/mL of Collagenase NB6 solution, respectively, to the labial salivary glands. We placed the tubes horizontally in a 37C incubator for 60 min, rotating at 60 rpm. After digestion we added 5 ml 1X DPBS to the cells. We strained cells through a 100-micron strainer, collecting the single cells to a 50 ml conical tube. We pelleted cells by centrifugation at 1500 rpm for 5 minutes at room temp. We decanted the supernatant, resuspend the cells in 1 ml culture media, add to a T25 flask containing 4 ml of media. We expanded and harvested our cells as described elsewhere.
